# Culicidae evolutionary history focusing on the Culicinae subfamily based on mitochondrial phylogenomics

**DOI:** 10.1038/s41598-020-74883-3

**Published:** 2020-11-02

**Authors:** Alexandre Freitas da Silva, Laís Ceschini Machado, Marcia Bicudo de Paula, Carla Júlia da Silva Pessoa Vieira, Roberta Vieira de Morais Bronzoni, Maria Alice Varjal de Melo Santos, Gabriel Luz Wallau

**Affiliations:** 1 Departamento de Entomologia, Instituto Aggeu Magalhães (IAM) - Fundação Oswaldo Cruz (FIOCRUZ), Recife, 50740-465 Brazil; 2 Faculdade de Saúde Pública, Universidade de São Paulo, São Paulo, 01246-904 Brazil; 3 Instituto de Ciências em Saúde, Universidade Federal de Mato Grosso, Sinop - MT, 78550-728 Brazil

**Keywords:** Evolutionary genetics, Evolutionary biology

## Abstract

Mosquitoes are insects of medical importance due their role as vectors of different pathogens to humans. There is a lack of information about the evolutionary history and phylogenetic positioning of the majority of mosquito species. Here we characterized the mitogenomes of mosquito species through low-coverage whole genome sequencing and data mining. A total of 37 draft mitogenomes of different species were assembled from which 16 are newly-sequenced species. We datamined additional 49 mosquito mitogenomes, and together with our 37 mitogenomes, we reconstructed the evolutionary history of 86 species including representatives from 15 genera and 7 tribes. Our results showed that most of the species clustered in clades with other members of their own genus with exception of *Aedes* genus which was paraphyletic. We confirmed the monophyletic status of the Mansoniini tribe including both *Coquillettidia* and *Mansonia* genus. The Aedeomyiini and Uranotaeniini were consistently recovered as basal to other tribes in the subfamily Culicinae, although the exact relationships among these tribes differed between analyses. These results demonstrate that low-coverage sequencing is effective to recover mitogenomes, establish phylogenetic knowledge and hence generate basic fundamental information that will help in the understanding of the role of these species as pathogen vectors.

## Introduction

Mosquitoes compose a large group of insects from the Culicidae family. There are around 3.567 valid species classified into two subfamilies (*Anophelinae* and *Culicinae*) and 41 genera (https://mosquito-taxonomic-inventory.info/ accessed on 21 Oct., 2019). The vast majority of mosquitoes species have anthropophilic behaviour towards reptiles and mammals including humans^1^. Because of that they can transmit many pathogens such as bacteria^2^, malaria protozoa^3^, filarial worms^4^ and arboviruses^5^ to the species they feed upon. Mosquitoes are responsible for the transmission of pathogens that cause outbreaks and epidemics annually in the tropical region, but the current globalization and land use change are increasing human-mosquito contact allowing the emergence of new mosquito-borne disease^6–8^. Several of the new emerging pathogens arose from forested environments where they circulate in a sylvatic cycle between wild animals and arthropod vector species such as mosquitoes^9^. Although there is abundant evidence that spillover occurs from sylvatic to urban environments, we know very little about the sylvatic cycle of these pathogens including the vector species that transmit them in their natural environment^10^. Therefore, basic knowledge about vector evolution and ecology is highly needed to better understand their role in the transmission cycle of pathogens^11–13^.

The huge improvement in nucleic acid sequencing platforms in the last decade has allowed an explosion of genomic information from a wide range of species. Mitogenomes, the entire mitochondrial genome, have been widely used as a target molecule to elucidate different aspects of metazoa species evolution such as population dynamics and phylogenetic relationships^14^. Complete mitogenomes are reliable tools to be used as a source of molecular markers in ecological and evolutionary studies because they provide genes with different evolutionary rates such as the most conserved rRNA genes (12S and 16S), the intermediate ND1-6 genes and the fast evolving cytochrome *c* oxidase subunit I (COI) gene, the most used molecular marker for species identification, allowing an accurate establishment of both ancient and recent speciation events^15–17^. In addition, mitogenomes have uniparental heritage, high copy number by cells and single-copy genes which facilitates DNA recovery and phylogenetic analysis^18–20^. Recently, some studies have sequenced a larger number of mitochondrial genomes from different mosquito species, but they are mostly focused on species from the *Anopheles* genus^21–23^. Mosquito mitogenomes are structurally conserved following the metazoa gene number and order, with few exceptions, showing 37 genes comprising 13 protein coding genes, 22 tRNAs and 2 rRNA genes^24–26^. Its size range varies from 14,820 bp for *An. maculatus* to 16,790 bp for *Ae. aegypti* (https://www.ncbi.nlm.nih.gov/genome/browse#!/organelles/culicidae accessed on 21 Oct, 2019).

Mitogenome sequencing has been a hard task using the first generation of sequence platforms based on the Sanger method. The first mosquito mitogenome was obtained after laborious steps such as mitochondria purification followed by DNA extraction, cloning and Sanger sequencing of several fragments^27,28^. Today there are a number of alternative approaches available to obtain mitogenomes which was only possible due to the improvement of the second and third generation sequencing platforms. Most of these strategies are based on PCR/Long Range PCR coupled with Next-generation sequencing (NGS), shotgun Whole Genome Sequencing or mitogenome sequencing through RNA-Seq data^29,30^. Other approaches available allow the recovery of mitogenomes by PCR amplification from environment samples and pooled DNA and mitogenome recovery from low-coverage sequencing^31–33^. Moreover, a number of bioinformatics tools were developed to specifically assembly and annotate mitogenomes^34–38^.

Most of the available mosquito mitogenomes belong to *Anopheles* species with fewer genomes for *Culex, Aedes* and other genera such as *Haemagogus, Bironella, Sabethes,* and *Lutzia*^21–23,26,39^, but there is no available molecular data for the large majority of the species. Aiming to contribute with this basic and fundamental knowledge we performed low-coverage whole genome sequencing and data mining on already published Culicidae SRA data to characterize the mitogenomes from different species and genera. Overall, we reconstructed and positioned 37 mitogenomes, 35 of them for the first time, representing 11 genera. Our evolutionary analysis covered a large diversity of the Culicinae subfamily evaluating 7 representatives from 11 existing tribes.

## Results

### Sequencing and mitogenome characterization

The sequenced mosquito samples generated a total of 84.2 million paired-end reads representing the 17 species and eight genera (*Aedeomyia, Aedes, Coquillettidia, Culex, Mansonia, Psorophora, Trichoprosopon* and *Uranotaenia*). The amount of generated reads ranged from 1.1 million reads for *Ur. pulcherrima* to 11.3 million reads for *Ae. taeniorhynchus* (Table 1). Searching on the SRA database, we included raw sequencing datasets of additional 20 mosquito species for mitogenome characterization representing six genera (*Aedes, Anopheles, Culex, Psorophora, Tripteroides* and *Toxorhynchites*). Here, we characterized 35 mitogenomes for the first time and reassembled the *An. aquasalis* and *Cx. nigripalpus* mitogenomes that were recently published^21,40^. In summary, the newly characterized mitogenomes represent eight Culicidae genera that had no mitochondrial genome data available to the best of our knowledge (*Aedeomyia, Coquillettidia, Mansonia, Psorophora, Trichoprosopon, Tripteroides, Toxorhynchites,* and *Uranotaenia*).

**Table 1:** General statistics for assembled draft mitogenomes.

Species	Total of reads (Mi)	Final assembly (bp)	Coverage breadth* (%)	Mapped reads^a^	Final coverage depth	% of mito reads	Total of genes	PCG	rRNA	tRNA
**Statistics for mitogenomes of species sampled and sequenced in this study**										
*Ae. taeniorhynchus*	11.3	14,732	95.10	8174	37.73	0.07234	37	13	2	22
*Ma. wilsoni*	8.2	10,443	67.41	3522	23.27	0.04295	28	12	2	14
*Tr. digitatum*	7.6	8282	53.46	729	5.99	0.00959	28	10	2	16
*Ae. scapularis*	7	7795	50.32	218	1.99	0.00311	25	11	1	13
*Ma. titillans*	6.6	11,181	72.18	7338	46.60	0.11118	27	11	2	14
*Cq. chrysonotum*	6.2	12,032	77.67	1955	11.37	0.03153	35	13	2	20
*Cq. juxtamansonia*	5.5	7711	49.78	826	7.18	0.01502	18	8	2	8
*Ps. cingulata*	4.9	15,660	101.09	3551	16.33	0.07247	36	13	2	21
*Cx. nigripalpus*	4.8	14,492	93.55	3633	17.30	0.07569	37	13	2	22
*Cq. venezuelensis*	4.3	13,912	89.81	2706	13.42	0.06293	34	13	2	19
*Ad. squamipennis*	3.9	10,466	67.56	899	5.93	0.02305	27	9	2	16
*Cx. corniger*	3.9	5222	33.71	137	1.84	0.00351	20	9	2	9
*Cx. amazonensis*	2.5	15,265	98.54	3274	15.87	0.13096	36	13	2	21
*Cq. albicosta*	2.4	14,689	94.82	26,487	115.40	1.10363	36	13	2	21
*Ma. humeralis*	2.2	3699	23.88	193	3.91	0.00877	14	7	2	5
*Cq. hermanoi*	1.8	12,289	79.33	1958	11.79	0.10878	26	10	2	14
*Ur. pulcherrima*	1.1	13,845	89.37	781	4.23	0.07100	32	12	2	18
Average	4.95	11,277	72.8	66,381	20.01	0.11450	–	–	–	–
**Statistics for mitogenomes assembled from SRA data**										
*Ae. alboannulatus*	45.6	14,314	92.40	2,645,908	22,749.53	5.80243	26	11	2	13
*Ae. camptorhynchus*	41	13,825	89.25	1,461,943	11,897.19	3.56571	30	12	2	16
*Ae. detritus*	83.6	14,707	94.94	2,228,562	19,597.21	2.66574	31	13	2	16
*Ae. fluviatilis*	6.2	14,360	92.70	3,22,138	3858.73	5.19577	17	12	1	4
*Ae. polynesiensis*	31.8	15,144	97.76	39,618	133.74	0.12458	34	12	2	20
*Ae. riversi*	17	5992	38.68	12,505	23.83	0.07356	17	10	1	6
*An. albimanus*	101.1	15,674	101.18	668,072	4304.92	0.66080	34	12	2	20
*An. aquasalis*	0.344	11,201	72.31	12,674	571.41	3.68002	14	9	2	3
*An. freeborni*	148.8	15,960	103.03	1,231,449	9200.00	0.82759	33	12	2	19
*An. nuneztovari*	76.9	12,741	82.25	42,462	446.26	0.05522	23	12	2	9
*An. quadriannulatus*	66.2	15,533	100.27	206,252	1341.11	0.31156	34	12	2	20
*Cx. australicus*	44.8	15,195	98.09	3,222,91	26,720.50	7.19395	32	13	2	17
*Cx. globocoxitus*	44.2	15,123	97.62	2,970,924	24,483.16	6.72155	31	12	2	17
*Cx. hortensis*	91.4	13,702	88.45	3,053,258	12,444.56	3.34054	25	11	2	12
*Cx. molestus*	27.2	11,440	73.85	558,460	4194.91	2.05316	21	13	2	6
*Cx. tarsalis*	37.8	15,831	102.19	3,037,171	28,602.31	8.03484	33	12	2	19
*Cx. torrentium*	47.8	12,652	81.67	558,460	4194.91	1.16833	26	13	2	11
*Ps.albipes*	135.6	15,791	101.94	1,087,575	8166.76	0.80205	33	12	2	19
*Tp. aranoides*	37.6	8990	58.03	287,439	4887.87	0.76447	9	7	2	0
*Tx. amboinensis*	100	15,775	101.83	2,220,264	14,215.32	2.22026	34	13	2	19
Average	59.24	13,698	88.2	1,293,401	5108	2.6311	–	–	–	–

*PCG * protein coding genes.

*Coverage breadth was calculated in relation to the average of culicidae mitochondrial genomes length (15,491 bp). Bp represents base pairs.

^a^Mapped *Reads* against final assembly using *MIRAbait* tool.

**Figure 1:**
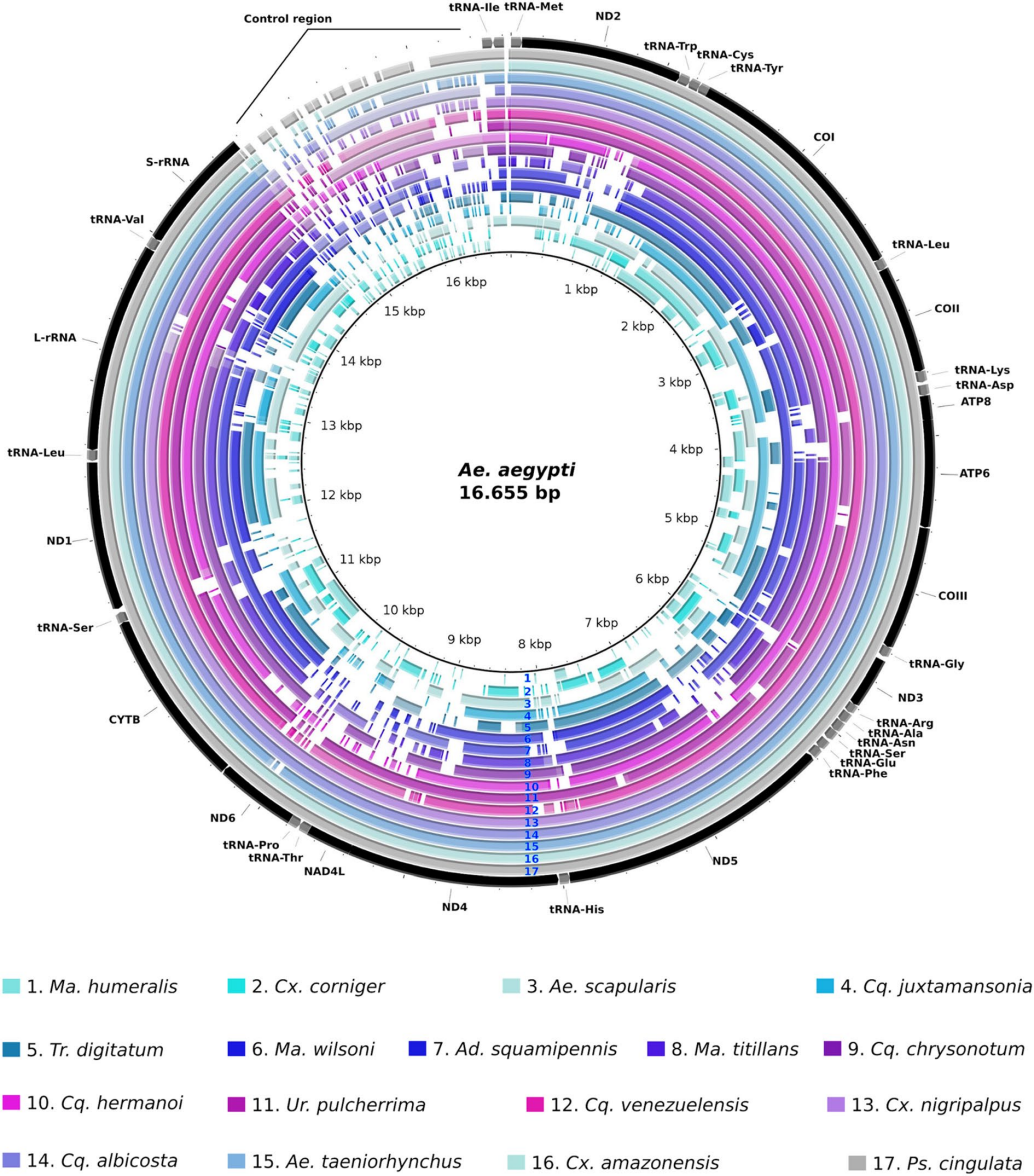
Comparative map of mitogenomes sequenced in relation to *Ae. aegypti* mitochondrial genome (NC_010241.1).

**Figure 2:**
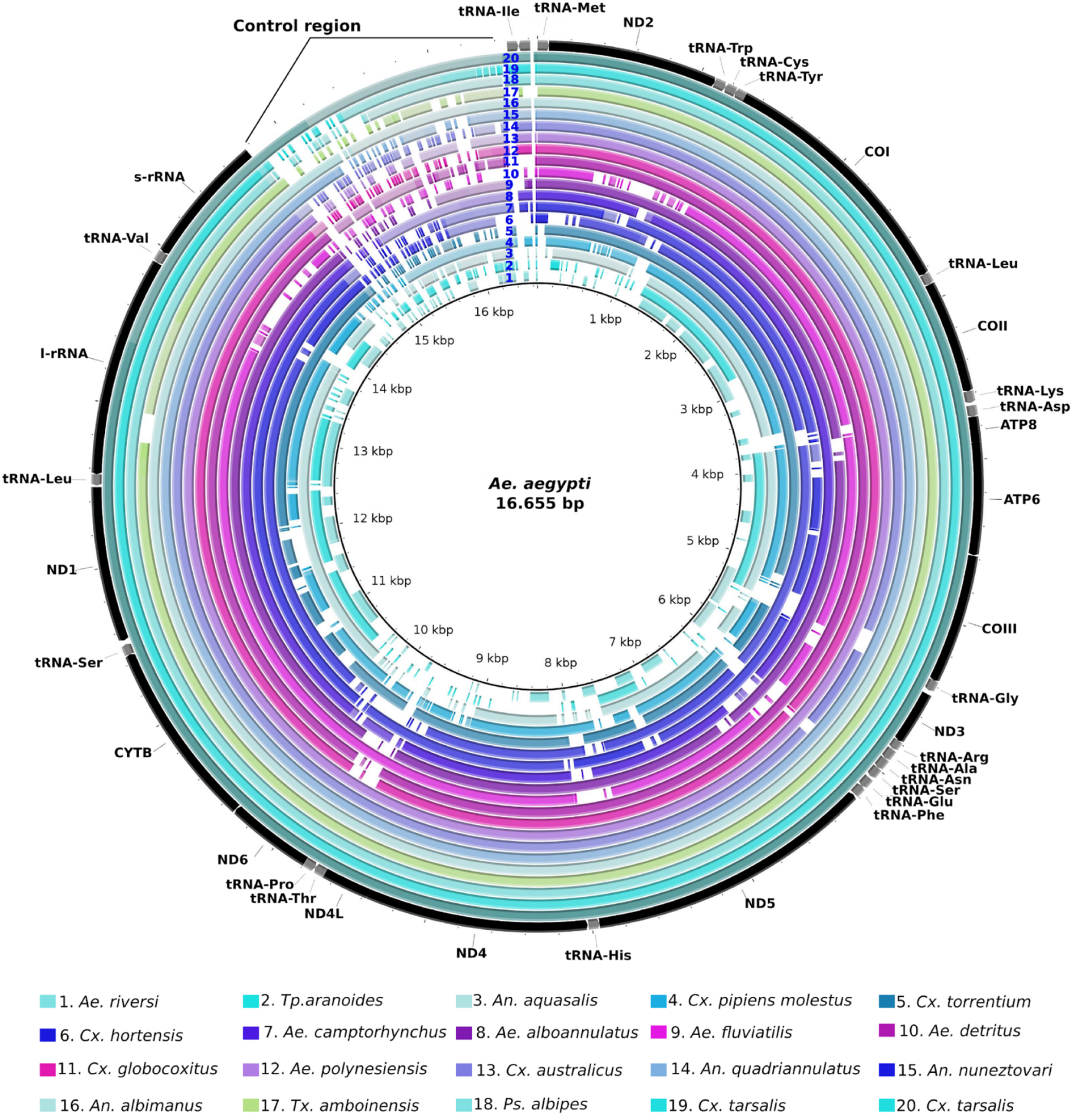
Comparative map of mitogenomes characterized from SRA data in relation to *Ae. aegypti* mitochondrial genome (NC_010241.1).

The coverage breadth of the sequenced draft mitogenomes ranged from 3699 to 15,660 bp for *Ma. humeralis* and *Ps. cingulata,* respectively (Table 1, Fig. 1) with an average coverage breadth of 72.80% and a coverage depth average of 20.01 fold (Table 1). Annotation of the protein coding genes (PCG) identified in the field-collected mosquitoes ranged from seven to 13. All 17 mitogenomes showed the two rRNA genes, except *Ae. scapularis* genome. In addition, tRNAs annotation ranged from five to 21 genes, except for *Ae. taeniorhynchus* and *Cx. nigripalpus* that showed all tRNAs genes (Table 1). Although some PCGs were not assembled, we could annotate the barcode COI in all 17 mitogenomes (Supplementary Table 1). The mitogenomes characterized from SRA data showed a coverage breadth ranging from 5992 to 15,960 bp for *Ae. riversi* and *An. freeborni* respectively (Fig. 2). In general those assemblies showed an average coverage breadth of 88.42% and from nine to 34 out of 37 mitochondrial genes were annotated with MITOS (Table 1). Although some of the SRA data came from RNA-Seq, we were able to identify almost all PCGs of these mosquito species. PCGs annotation ranged from seven for *Tp. aranoides* to 13 for other species (Supplementary Table 1, Fig. 2).

### Evolutionary analysis

**Figure 3:**
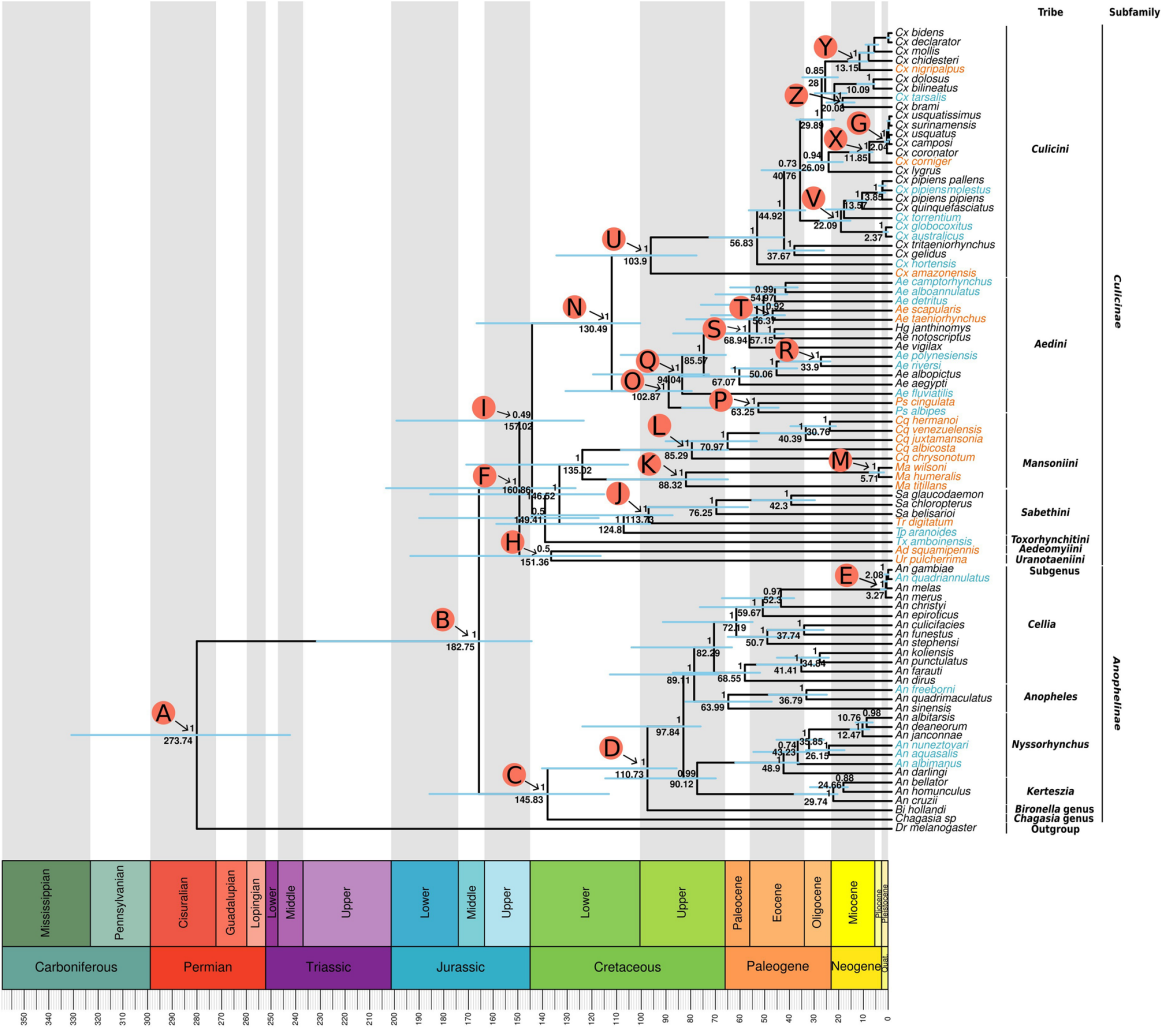
Evolutionary timescale of Culicidae family. Tree was generated from BEAST using PCGs nucleotide sequences partitioned by gene and codon positions (1st + 2nd and 3rd separately). Blue bars represent the HPD95%. The numbers above and below the bars show the posterior probability and the predicted median dating respectively for each node. Specific words inside the circles represent the nodes discussed in the text. Light blue tip names represent mitogenomes characterized from SRA data. Orange tip names represent sequenced mitogenomes from this study.

**Figure 4:**
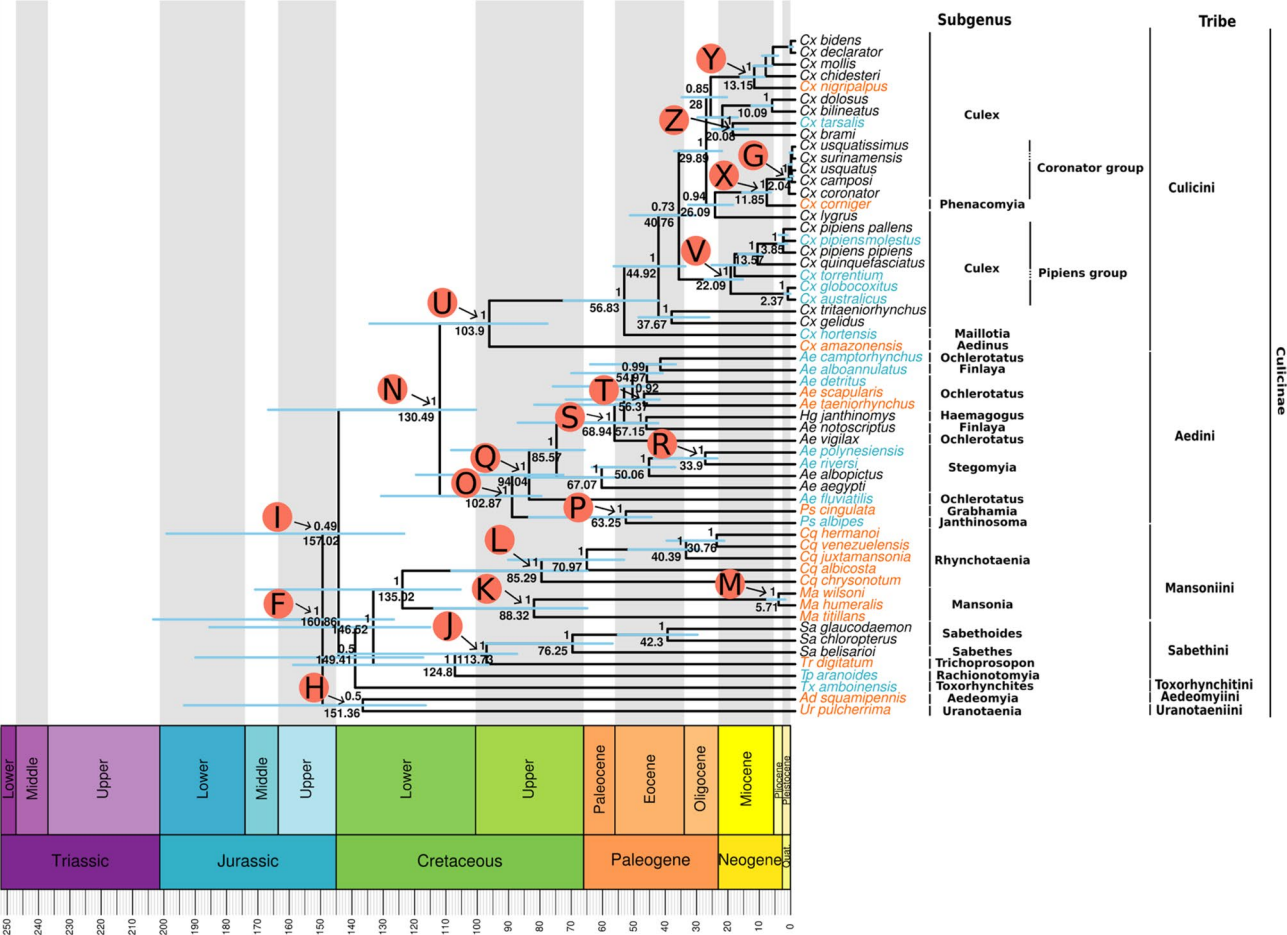
Zoom on the Culicinae branch of Fig. 3 showing in more detail the evolutionary timescale of Culicinae subfamily. Tree was generated from BEAST analysis of partitioned PCG taking into account the split of codon positions (1st + 2nd and 3rd codon position separately). Blue bars in the nodes represent the HPD95%. The numbers above and below the bars show the posterior probability and the predicted median dating respectively for each node. Specific words inside the circles represent the nodes discussed in the text. Light blue tip names represent mitogenomes characterized from SRA data. Orange tip names represent sequenced mitogenomes from this study.

**Figure 5:**
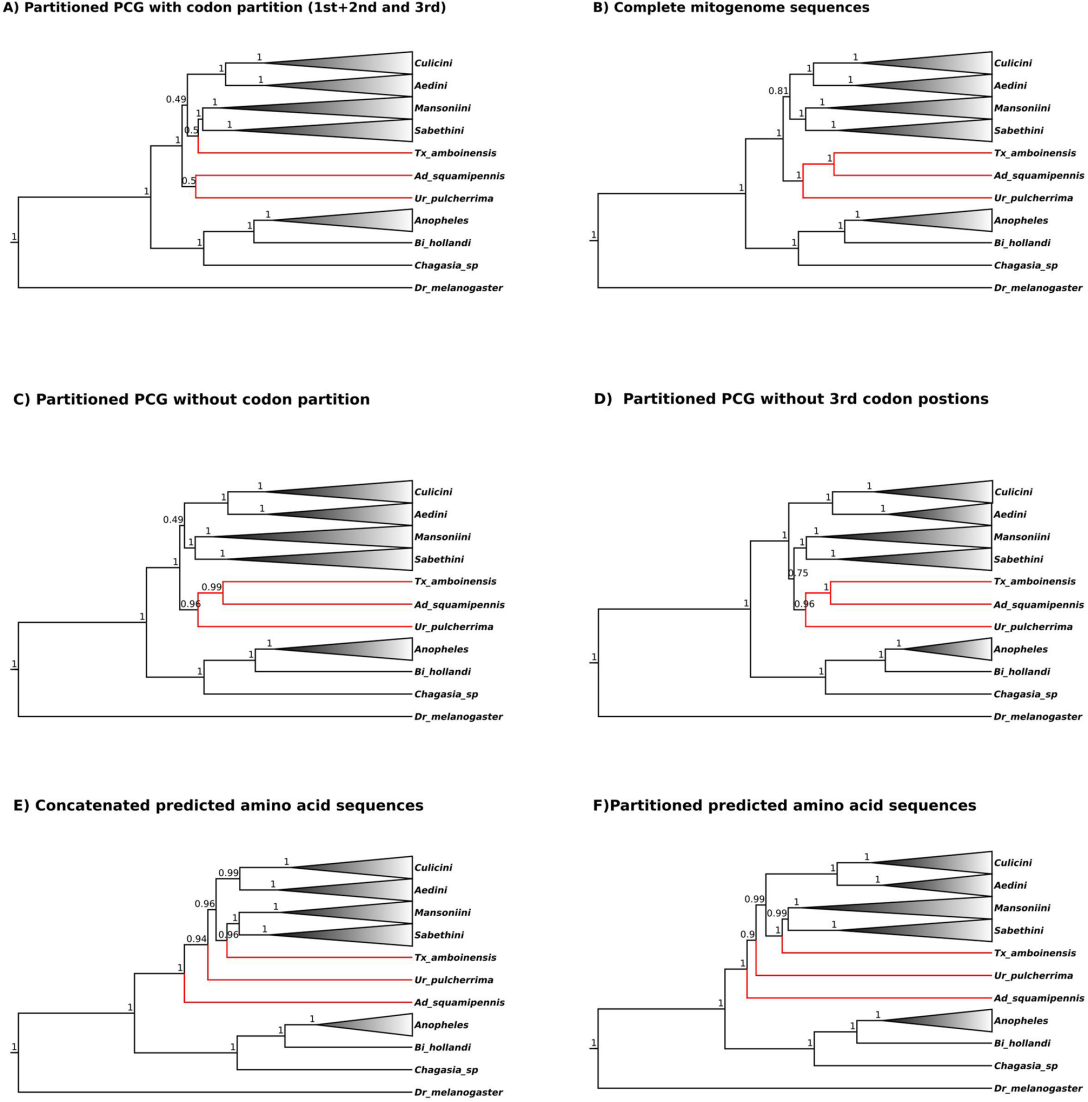
Bayesian inferred trees showing the incongruences in positioning of *Aedeomyia, Uranotaenia* and *Toxorhynchites* genera using different alignment datasets.

In order to establish the phylogenetic relationship of the Culicidae family we performed the phylogenetic analysis of 86 different mosquito mitogenomes representing 15 genera, including our 35 newly characterized mitogenomes. The analysis was based on nucleotide and amino acid datasets with or without partitioning. Since several genes showed nucleotide saturation at the third codon position (Supplementary file 1) we also performed phylogenetic analyses with codon partitions of each PCG and without the third codon positions (Supplementary Figs. 3–10). Topology of the phylogenetic trees built with those different alignments were mostly in agreement, but incongruences and variable positioning of some deep branches was observed for (*Culicini* + *Aedini*) + (*Mansoniini* + *Sabethini*) tribes and *Aedeomyia, Uranotaenia* and *Toxorhynchites* species (Figs. 3, 4 and 5 and Supplementary Fig. 1 and 2). In respect of the (*Culicini* + *Aedini*) + (*Mansoniini* + *Sabethini*) recovered relationship: using partitioned PCG taking or not into consideration the partitioning codon position showed a low posterior probability branch support of (0.49—Figs. 3, 4 and 5A,C), while complete mitochondrial genomes and concatenated and partitioned amino acid sequences supported this same clade with high branch support (0.81, 0.96, 0.99, respectively—Fig. 5B,E,F). Only the partitioned PCG without 3rd codon positions showed a different grouping with *Mansoniini* + *Sabethini* as a sister clade of (*Tx. amboiensis* + *Ad. squamipennis*) + *Ur. pulcherrima,* but with a relatively low posterior probability support (0.75). Regarding the variable positioning of *Tx. amboinensis* species, it was placed either as a basal clade of *Mansoniini* and *Sabethini* tribes in partitioned PCG taking into consideration (1st + 2nd and 3rd) codon positions but with a low posterior probability support value (0.5—Figs. 4, 5A) and concatenated and partitioned amino acid sequences with high branch support (0.96 and 1.0, Fig. 5E,F) or forming a clade with *Ad. squamipennis* and *Ur. pulcherrima*—(*Tx. amboiensis* + *Ad. squamipennis*) + *Ur. pulcherrima*—in complete mitochondrial genome tree (Fig. 5B, posterior probability of 1), partitioned PCG without codon partition (Fig. 5C, posterior probability of 0.99 and 0.96), partitioned PCG without 3rd codon position (Fig. 5D, posterior probability of 1 and 0.96). Moreover, a number of intra genus incongruences between the trees was observed in the *Culex, Anopheles,* and *Aedes* genera (Supplementary Fig. 1 and 2).

The evolutionary timescale of the Culicidae family showed the radiation of the last common ancestral between mosquitoes and *Drosophila* occurring during the Permian period around 273 million years ago (MYA) (Fig. 3, node A and Supplementary table 2). While the most recent common ancestor of the Culicidae family emerged in the Jurassic period around 182 MYA with the *Anophelinae* and *Culicinae* subfamilies origin (Fig. 3, node B). In the *Anophelinae* subfamily*,* the *Chagasia* genus was basal to *Bironella* and *Anopheles* genera with speciation in the Cretaceous period around 145 MYA (Fig. 3, node C). The last two genera showed speciation times from 110 to two MYA in the *gambiae* species complex (Fig. 3, nodes D and E, respectively).

The *Culicinae* subfamily formed a monophyletic group with the TMRCA (The most recent common ancestral) occurring around 160 MYA in the Jurassic period (Fig. 3, node F). Among *Sabethini* members, the *Tripteroides* genus was positioned as a basal lineage and the *Trichoprosopon* genus splited from other *Sabethini* species around 113 MYA (Fig. 4, node J). The *Mansoniini* tribe was placed as a sister clade to the *Sabethini* tribe with high posterior probability branch support (1.0) in all phylogenetic analysis performed (Supplementary Fig. 3–10). The *Mansonia* and *Coquillettidia* genera were both monophyletic with speciation processes starting around 88 and 85 MYA respectively (Fig. 4, nodes K and L, respectively).

The diversification between *Culex* and *Aedini* taxa occurred in the Cretaceous period around 130 MYA (Fig. 4, node N). While the split of *Aedes* and *Psorophora* genera occurred around 102 MYA, and the speciation of *Ps. albipes* and *Ps. cingulata* occurred in Paleogene around 63 MYA (Fig. 4, node O and P, respectively). Among the *Aedes* species, *Ae. fluviatilis* was recovered as the basal and early diverged species (94 MYA, node Q in Fig. 4) from the genus in eight out of nine phylogenetic reconstructions performed (Fig. 4 and Supplementary Fig. 3–10). *Ae. polynesiensis* and *Ae. riversi* were close to *Ae. albopictus* (Fig. 4, node R). Another clade formed closely to *Ae. aegypti* clade was composed by species from *Ochlerotatus* (*Ae. vigilax, Ae. taeniorhynchus, Ae. scapularis, Ae. detritus,* and *Ae. camptorhynchus*), *Finlaya* subgenera (*Ae. notoscriptus* and *Ae. alboannulatus*), and *Haemagogus* genus, in which *Ae. vigilax* was the basal species (Fig. 4, node S). The *Finlaya* subgenus has a paraphyletic status when the positioning of *Ae. alboannulatus* and *Ae. notoscriptus* is observed (Fig. 4). The neotropical species *Ae. taeniorhynchus* and *Ae. scapularis* formed a clade and diverged between themselves around 56 MYA (Fig. 4, node T)*.* Among *Culex* species, *Cx. amazonensis* a member of the subgenus *Aedinus*, showed to be the basal and the earlier diverged species from the genus with the split from the other species occurring around 103 MYA (Fig. 4, node U). The *pipiens* group, from *Culex* subgenus, originated around 22 MYA in which the Australian species *Cx. australicus* and *Cx. globocoxitus* were placed in basal position in relationship to other *Cx. pipiens* species (Fig. 4, node V). *Cx. torrentium* grouped in the *pipiens* group. *Cx. corniger,* a member of *Phenacomyia* subgenus, was a basal species to the coronator group from *Culex* subgenus (node G), as well as, *Cx. nigripalpus* that speciated from *Cx. chidesteri, Cx. mollis, Cx. declarator* and *Cx. bidens* (Fig. 4, nodes X and Y, respectively). Moreover, *Cx. tarsalis* formed a clade with *Cx. brami* (Fig. 4, node Z).

## Discussion

Mitogenomes have been widely used to elucidate the evolutionary history of several species of animals and plants and can also be used as barcode sequences for species identification^16,41,42^. The first mosquito mitogenome from *An. gambiae* was sequenced using the Sanger method on PCR amplified fragments^27^ and further mitochondrial genomes were slowly sequenced along with whole genome projects. Currently, most studies have been using whole genome sequencing or PCR amplification followed by high throughput sequencing to characterize several mitogenomes at once^22,23,26^ in a wide range of insect species showing promising results to reconstruct mitogenomes^32,43^. Here, we performed low-coverage whole genome sequencing to assemble and characterize the mitogenomes from seventeen mosquito species. We were able to generate 16 new draft mitogenomes from Culicidae species belonging to eight different genera. This approach has already been used in other studies demonstrating a cost-effective way to recover mitogenomes for evolutionary studies^32,43–46^. Richter et al. (2015)^32^ suggested that a minimum of 10 million reads are needed to recover mitogenomes with higher coverage breadth and datasets having around 1 million reads usually generate highly incomplete mitogenomes. Even though our study used different organisms and algorithm to obtain the mitogenomes in comparison to Richter et al. (2015)^32^, we were able to assemble nearly complete mitogenomes with as low as 1.1 million reads (Table 1). Besides, our draft mitogenomes assembled contained enough phylogenetic markers necessary for robust phylogenetic analysis.

Additionally, we reconstructed mitochondrial genomes from available RNA-Seq data. We were able to reconstruct 19 additional nearly complete draft mitogenomes for Culicidae species that had no mitogenomes available. No study has been able to reconstruct complete mitochondrial genomes from RNA-Seq data, mainly due to the endonuclease activity on transcripts or loss of mitochondrial transcripts due to the enrichment steps normally used during the sequencing library construction^30^. However, the remaining mitochondrial data available in different RNA-Seq datasets may still be used to retrieve mitogenomic sequences^47–49^. The datasets used for mitogenomes characterization contained around 0.073 to 8.034% of mitochondrial reads. In total, we were able to assemble 37 draft mitogenomes in this study representing 11 genera (*Anopheles, Uranotaenia, Aedeomyia, Toxorhynchites, Tripteroides, Trichoprosopon, Mansonia, Coquillettidia, Psorophora, Aedes,* and *Culex*)*.*

Several efforts have been made to better understand the taxonomic status of groups inside of the Culicidae family, but most studies that included a substantial number of species employed only morphological data^50^ and the ones using molecular information suffer from limited sampling/taxonomic breadth^12,21–23,51–53^ and those with limited molecular markers^54^. Hence, there are still many non-studied species and unresolved phylogenetic relationships in genera such as *Aedes, Armigeres, Coquillettidia, Culex, Mansonia, Mimomyia, Psorophora, Topomyia, Tripteroides, Toxorhynchites, Uranotaenia,* and *Wyeomyia*^50^.

The phylogenetic analysis, including the 37 new mitogenomes assembled in this study comprising 11 Culicidae genera, is highly congruent regarding the monophyly of large species groups. Culicinae and Anophelinae subfamilies and *Anopheles, Sabethes*, *Mansonia, Coquillettidia*, *Psorophora,* and *Culex* genera were monophyletic. Moreover, we observed similar dating estimates as reported in the literature, for some key ancestors. For instance, our estimates of the common ancestor of Drosophila and Culicidae were around 273 MYA (HPD95%: 243.79–332.41), while other studies suggested that the common ancestor existed around 259 and 260 MYA using mitogenomes and phylogenomics analysis respectively^13,53^. The ancestral of *Anophelinae* and *Culicinae* subfamilies occurred in the Jurassic period around 182 MYA (HPD95%: 145.88–232.95). Similar estimates were obtained in other studies around 190–195 MYA^55,56^. Different evolutionary rates of molecular markers, limited species sampling and different algorithms used to reconstruct the species phylogeny could result in different time estimates^13^.

The evolutionary history of the *Anophelinae* subfamily has been more extensively studied considering the number of species analyzed, the morphological and molecular markers used including whole phylogenomic analysis^52^. A recent study using the mitochondrial genomes from several species, proposed a number of taxonomic status changes such as the elevation of some groups (*Cellia, Anopheles, Kerteszia* and *Nyssorhynchus*) from the subgenus to genus level^21^. Our results corroborate the monophyly of *Cellia, Anopheles, Kerteszia* and *Nyssorhynchus* subgenera although, our sampling of the *Anopheles* genus is insufficient to directly compare with Foster and collaborators^21^ on whether Anopheles should be split into multiple genera. Our phylogenetic analysis diverged from Foster’s study^21^ regarding the positioning of *Kertezia* subgenus that was sister group of *Nyssorhynchus* (Fig. 3) while Foster et al. (2017)^21^ recovered an uncertain grouping of *Kertezia* with other *Anophelinae* subgenera. In addition, Foster et al. (2017)^21^ and Neafsey et al. (2015)^52^ have not assessed the temporal diversification of some basal groups such as *Chagasia* and *Bironella*. In our analysis, these groups showed to be the early diverged lineages from *Anophelinae* subfamily, emerging in the Upper and Lower Cretaceous, respectively (Fig. 3) and *Bironella* genus showed to be an ancestral lineage in relation to *Anopheles* genus, including all *Anopheles* subgenera assessed in our study such as *Kerteszia*, *Nyssorhynchus, Anopheles,* and *Cellia.* Those results contrast with Foster et al. (2017)^21^ regarding *Bironella* positioning that suggest it grouped within *Anopheles* genus but with a low branch support. Previous studies, using both nuclear ribosomal sequences and fragments of mitochondrial genes COI and COII of *Bi. gracilis*^57^ and *Bi. hollandi*^58^, have already suggested the positioning of *Bironella* within the *Anopheles* genus. Although the number of the species analyzed, different molecular markers and phylogenetic approaches used in these studies, these contrasting results show that *Bironella* genus position and phyletic status are still open and a wide sampling of the genus and molecular markers are needed to uncover it. Regarding the *Anopheles* species, our analysis using mitogenomes showed a similar positioning as previously presented in other studies^12,13,40,58–60^.

The radiation in the *Culicinae* subfamily is older than *Anophelinae* around 160 MYA (HPD95%: 128.09–204.91) in the Jurassic period (Supplementary Fig. 11). In the *Culicinae* subfamily, we detected three low supported deep branch clades in the partitioned PCG taking into account the split of codon positions (Fig. 4), the *Ad. squamipennis* + *Ur. pulcherrima* (node H)*,* the (*Culicini* + *Aedini*) + *(Mansoniini* + *Sabethini*) (node I) and the *Tx. amboinensis* basal positioning to *Mansoniini* + *Sabethini* clade. Regarding node I, there are substantial evidence in the literature corroborating the (*Culicini* + *Aedini*) + *Sabethini* grouping^26,61^ and all our complementary eight phylogenetic reconstructions using maximum likelihood and bayesian inference recovered this clade including *Mansoniini* as a sister group of *Sabethini* tribe (Fig. 5 and Supplementary Fig. 3–10). On the other hand, we found different results on the deep branching patterns and branch support for *Ad. squamipennis, Ur. pulcherrima* and *Tx. amboinensis* mainly depending on the molecule (nucleic acid or amino acid) used for phylogenetic reconstruction (Fig. 5)*.* In short, amino acid based reconstruction placed *Ad. squamipennis* as the earliest divergent species followed by *Ur. pulcherrima* and *Tx. amboinensis* as an early divergent clade of *Mansoniini* + *Sabethini* group with all high branch support, while nucleic acid based reconstruction show a more variable branching pattern grouping these tree species in a highly supported basal clade to the remaining *Culicinae* species or with lower support as a sister clade of *Mansoniini* + *Sabethini* species (Fig. 5B–D). Other studies, based on morphological characters, suggested that *Aedeomyia, Uranotaenia* and *Toxorhynchites* genera are ancient and basal groups in the *Culicinae* subfamily^62–65^ suggesting that our phylogenetic reconstruction based on amino acid sequences may have recovered the true position of those genera. This is in line with the widespread knowledge that conserved amino acid sequences are more appropriate to recover deep branching patterns^21,66,67^, but due to the high branch support of a clade encompassing the three studied species from these genera in the nucleotide based trees (Fig. 5) and two studies based on six nuclear genes and 18S rDNA have shown the positioning of *Ur. sapphirina* more closely related to *Culicini* and *Aedini* tribes, respectively^54,68^, additional phylogenetic analysis including more species and nuclear molecular markers will be needed to test the hypothesis raised in our study.

Regarding the *Sabethini* tribe, our results are in line with previous works that showed the monophyly of tribe, the basal positioning of *Tripteroides* (*Tp. aranoides*), and the sister positioning of *Trichoprosopon* genus (*Tr. digitatum*)^22,26,54^ (Fig. 4). Up to now, few studies have investigated the phylogenetic positioning and speciation time of the *Mansoniini* species. In our analysis, the eight species from the *Mansoniini* tribe formed a monophyletic group that is a sister group of the *Sabethini* tribe with a high posterior probability node support (1.0—Figs. 3, 4). Our results are in contrast with Reidenbach et al. (2009)^54^ analysis that positioned a single *Coquillettidia* species, *Cq. pertubans*, as a sister group of *Aedini* with a low posterior probability node support of 0.61*.* Our dataset covers a larger number of species from the *Mansoniini* tribe and more molecular markers than in Reidenbach’s study^54^, besides their study did not include any *Mansonia* species, which likely explain those differences.

Considering the *Aedini* tribe, our results showed the same basal positioning of *Psorophora* genus as observed by Reidenbach et al. (2009)^54^. Regarding *Aedes* genus, our results showed a paraphyletic group encompassing a single species from the *Haemagogus* genus which corroborates other findings with a larger number of *Haemagogus* species^69^. Besides, paraphyletic groups were observed for *Ochlerotatus* (*Ae. fluviatilis, Ae. taeniorhynchus*, *Ae. scapularis, Ae. vigilax, Ae. detritus,* and *Ae. camptorhynchus*) and *Finlaya* subgenus (*Ae. notoscriptus,* and *Ae. alboannulatus*), while *Stegomyia* subgenus (*Ae. aegypti, Ae. albopictus, Ae. riversi,* and *Ae. polynesiensis*) formed a monophyletic group (Fig. 4). A previous study, based on morphological cladistic analysis, suggested the monophyly of *Ochlerotatus* and *Finlaya* subgenera^70^. Inside of the *Aedes* genus, *Ae. fluviatilis* is the earliest branch in contrast to other studies that positioned it within *Aedes* branches^69^. Depending on the classification this species it is a member of Georgecraigius or Ochlerotatus taxa^71–73^, the basal positioning in our analysis renders Ochlerotatus group proposed by Reinert (2000)^72^, using morphological characters and supported by others^74^, non-monophyletic. In summary, the *Aedes* genus is a paraphyletic group showing several phylogenetic incongruences even considering studies that used different markers and species representatives. Hence, further reclassification is needed following the current knowledge of phylogenetic relationships of these species.

Regarding the *Culex* genus, our analysis showed that *Cx. amazonensis* and *Cx. hortensis* are the earliest diverged species from this genus. Our results are in agreement with Harbach’s. 2012^75^ cladistic morphological analysis concerning the basal positioning of these species, however our mitogenomic data support *Cx. amazonensis* as the earliest divergent species instead of *Cx. hortensis*. Our analysis placed *Cx. nigripalpus* as a sister group of the clade composed by *Cx. chidesteri, Cx. mollis, Cx. declarator,* and *Cx. bidens*, while, *Cx. corniger* was placed as a sister lineage of the *Coronator* group. A previous study using a fragment of the COI gene, has already suggested this positioning^76^ and our mitogenomic analysis supported this placement. It has been discussed, if *Cx. pipiens* consist in a species or a group of sibling species of *Pipiens* group^77^. Some authors describe the *Pipiens* group harboring the following species: *Cx. pipiens pipiens, Cx. quinquefasciatus, Cx. pipiens pallens, Cx. pipiens molestus, Cx. australicus,* and *Cx. globocoxitus*^77,78^. Other similar species such as *Cx. torrentium,* has not been considered as a member of *Pipiens* group due to its genetic divergence to other species of the group^79^. A study based on analysis of ITS1 and ITS2 has already demonstrated the close relationship of *Cx. torrentium* with *Pipiens* group^80^. Our analysis have positioned *Cx. torrentium* within *Pipiens* group with Australian members *Cx. globocoxitus* and *Cx. australicus* as basal clade, suggesting that *Cx. torrentium* may be a true species from the *Pipiens* group. Although the lower divergence time among some members of the Pipiens group each “species” has specific ecological, physiological and behavioral characteristics^79,81^.

## Conclusion

Overall, we characterized the phylogenetic position and speciation time of the main groups of the Culicidae family which emerged in the last 182 MYA between the Jurassic and Paleogene periods. Most of the different genera emerged in this range of time, but some recent speciation occurred in the *Culex* genus. Interestingly, a burst in mammals speciation also occurred in the Neogene period likely driving the speciation of these species at that time^55,82^. Furthermore, the new phylogenetic knowledge will allow us to propose new hypotheses about some mosquito traits emergence and maintenance related with vector competence. More in depth studies trying to tease apart different molecular mechanisms of vector competence considering the phylogeny of the Culicidae tree will benefit from the information generated in this work.

## Material and methods

### Mosquito sampling and taxonomic identification

Mosquito samples were collected in remnants of the Brazilian Atlantic forest and from the South border of the Brazilian Amazon forest. Three municipalities were sampled in the Brazilian Atlantic forest, state of Pernambuco: Recife, at the Parque Estadual Dois Irmãos (8°00′43.3″S 34°56′40.7″W); Moreno, at the Reserva Ecológica de Carnijó (8°08′20.7″S 35°04′47.3″W) and Camaragibe, at Aldeia (7°54′18.0″S 35°04′34.3″W). Three municipalities were sampled in the Brazilian Amazon forest, state of Mato Grosso: Sinop (− 12°04′73.9″S − 55°43′85.0″W); Sorriso (− 12°16′85.9″S − 55°70′68.3″W); and Ipiranga do Norte (− 11°61′08.2″S − 55°73′41.7″W). Different sampling methods were employed aiming to collect a large diversity of species. Diurnal sampling were performed with aspirators (HORST model) and entomological nets, larvae and pupae were collected on water pools and plant holes. Nocturnal sampling were performed using CDC-light traps and BG-Sentinel to sample mosquitoes attracted by light and odorants. The specimens were transported alive either to the Entomology department of Aggeu Magalhães Institute—Oswaldo Cruz Foundation (IAM/FIOCRUZ) or to the Molecular Biology and Immunology Laboratory—Federal University of Mato Grosso (LIBM/UFMT). Immature specimens were maintained in liquid water and fed with cat food (FRISKIES) until the emergence of adults. Adult mosquitoes were separated into morphological groups and dry stored in silica at room temperature until taxonomic identification. Taxonomic keys for neotropical Culicidae were used for species identification^83,84^. Besides the collection performed in this work, we included *Ae. taeniorhynchus* and *Ae. scapularis* samples provided by collaborators of the Entomology department of IAM, sampled respectively in the municipality of São Luis, state of Maranhão and in municipality of Juazeiro, state of Bahia. All collections were authorized by the regulatory organ—SISBIO under the license numbers: 58716-1 and 47284-2.

### DNA extraction and sequencing

The specimens were macerated in ultrapure water using 40ul/specimen in single or pooled samples (Supplementary table 3) according to the number of specimens collected per species. Both male and female individuals from different collection points were included in the pools. Total DNA extractions were performed either by ethanol precipitation method^85^ or QIAprep Spin Miniprep extraction (QIAGEN) in order to improve mitochondrial DNA by enrichment as suggested by Quispe-Tintaya et al. (2013)^86^. All samples were assessed by quality and purity with NanoDrop 2000 (THERMO SCIENTIFIC) and quantified through Qubit dsDNA HS (High Sensitivity Assay) kit. The DNA library was prepared using the Nextera XT library preparation kit following the recommendations of the manufacturer (ILLUMINA, San Diego, CA, USA). DNA library was sequenced using a low-coverage whole genome sequencing strategy using the ILLUMINA Miseq platform. We employed a paired-end approach of 75 bases with Reagent Kit V3 of 150 cycles.

### Dataset construction

A search on the National Center for Biotechnology Information (NCBI) was performed to recover previously characterized mitochondrial genomes from *Culicinae* and a subset of *Anophelinae* subfamily representing different genera comprising 50 mitogenomes (Supplementary table 4). Besides, we searched on the SRA database for mosquitoes raw sequence reads (Whole genome sequencing and RNA-Seq) available up to November, 2018, representing species that had no mitogenome available at that time (Supplementary table 5).

### Quality control of sequences

The raw reads (sequenced in this study and recovered from SRA) were checked for quality using FastQC program (https://www.bioinformatics.babraham.ac.uk/projects/fastqc/ accessed on 21 Oct, 2019) and results were summarized on MultiQC tool^87^. Based on the excellent quality of our sequenced raw reads they were not trimmed (Supplementary Fig. 12) but, all SRA libraries were trimmed using the Trimmomatic tool v 0.35^88^ to remove adapters and ensure the quality of sequences (Phred score > 20).

### Mitogenome assembly and annotation

The mitogenomes were assembled using a baiting and iterative mapping approach implemented in MITObim 1.9^36^. Different mosquito mitogenomes were used as reference genome such as *Ae. vigilax, Ae. aegypti, Sa. belisarioi, Cx. quinquefasciatus* (accession numbers can be found in Supplementary table 4) for the first capturing of reads considering the closest mitogenome available to each species analyzed. SRA reads were assembled using MITObim default parameters (*-kbait* parameter = 31). Also, we used a combination of parameters to generate a consensus sequence for the sequenced species. A first assembly was performed using -*kbait* = 15 followed by a second assembly step using -*kbait* = 31. The final consensus assembly was composed by the consensus of the two assemblies, which was then checked with well characterized mitogenomes to correct any potential assembly errors (e.g. the assembly of non alignable regions between mitogenomes). To assess the average coverage depth of each mitogenome, the reads were mapped against the assembled mitogenomes through the *MIRAbait* module from MIRA sequence assembler software^89^.

Complete nucleotide sequences of the mitogenomes characterized in this study, were aligned by MAFFT v 7.0 tool^90^ with previously characterized complete mitogenomes recovered from databases (Supplementary table 4 and Supplementary file 2). The non-aligned sites were removed using GBLOCKS tool v. 0.91b—default parameters, with exception for the allowed gap positions that was set with the “half” option^91^, to generate the final version of the mitochondrial genomes. Automatic gene annotation of the mitochondrial genomes were performed on MITOS2 web server (https://mitos2.bioinf.uni-leipzig.de/index.py accessed on 5 Dec., 2018)^37^ based on invertebrate genetic code against the metazoan Refseq 81. Comparative genomic maps were built using *Ae. aegypti* mitogenome (Accession number: NC_010241.1) as reference in BRIG (BLAST *Ring Image Generator*)^92^.

### Evolutionary analysis

Evolutionary analysis were performed based on five possible alignment approaches: (I) complete nucleotide mitogenome alignment sequences, (II) partitioned nucleotide sequence of protein coding genes derived from complete and draft mitogenomes with partitioned codon positions (1st + 2nd and 3rd), (III) partitioned nucleotide sequence of protein coding genes without 3rd codon positions without codon partitioning, (IV) partitioned predicted amino acid sequences from coding regions and (V) concatenated alignment of amino acid sequences. Final alignment was visualized and checked on Aliview^93^. Nucleotide substitution saturation analysis was performed for each nucleotide gene alignment in DAMBE software^94^ evaluating 1st + 2nd and 3rd codon position separately through the Xia et al. test^95^. Nucleotide substitution models for I, II and III alignments were obtained with Smart model selection (SMS) implemented on PhyML webserver^96^. Protein evolutionary models were assessed for IV and V alignments using Prottest 3.4.2^97^. All divergence dating analysis were based on a Bayesian Markov Monte Carlo approach (MCMC) performed on BEAST 1.8.4 package^98^ to infer the topology of Culicidae family and the speciation time of the common ancestor of clades in million years. A previous literature search was performed to obtain fossil dates representing the different Culicidae clades and calibrate the molecular clock analysis. Although there are several potential calibration points to the Culicidae tree we only kept the ones supported by fossil evidence. We used four calibration points representing the Diptera order, Culicidae family and Anophelinae and Culicinae subfamily (Supplementary table 6 and Supplementary Fig. 13).

Bayesian analysis was performed with at least three independent runs of 150 million generations sampling at each 1000 trees, for each alignment dataset. The effective sample size of each parameter (ESS) was evaluated by Tracer 1.7.1^99^ and reached 200 for most of the important parameters for dating and tree likelihood. The analysis was performed under an uncorrelated relaxed molecular clock using a lognormal distribution and a Birth–Death model process of speciation as Tree Prior. For the complete mitochondrial genome alignment (alignment I) the GTR + G + I evolutionary model was used. For the partitioned gene analysis (alignment II) and partitioned predicted amino acids (alignment IV) each partition was set with a specific evolutionary model as previously described (Supplementary table 7). Besides, the partitioned gene analysis we also performed a more robust analysis based on the nucleotide saturation of each gene taking into account the codon position partitioning where the 1st and 2nd codon positions were split from the 3rd codon position. The concatenated protein analysis was performed under the mtREV + G + I evolutionary model. The posterior probability tree for each alignment dataset was built combining the three independent runs of each analysis with the LogCombiner program applying a burn-in of 25% and the consensus credible tree was obtained through the TreeAnnotator program. The timescale trees were plotted with Phyloch package version 1.5–3 (available on https://www.christophheibl.de/Rpackages.html accessed on 21 Oct, 2019) from R programming language. Tree topologies comparison were performed by plotting tanglegrams using the Dendextend R package^100^ based on trees obtained from BEAST analysis. Besides the bayesian analysis we also performed maximum likelihood phylogenetic analysis based on alignment I, III (concatenating individual alignments). The best evolutionary model was selected by the ModelFinder^101^ followed by the tree reconstruction using the IQ-TREE version 1.6.12^102^ performing the ultrafast bootstrapping^103^ with 1000 replicates. The consensus trees were visualized and edited on FigTree version 1.4.2 (available on https://tree.bio.ed.ac.uk/software/figtree/ accessed on 3 Jun, 2020).

## Supplementary information

Supplementary Information.

## Data Availability

Raw mitochondrial reads were submitted to European Bioinformatic Institute under the project number: PRJEB36702. The final mitogenome assemblies and raw phylogenetic tree files generated are available on Supplementary file 2 and Supplementary file 3, respectively (https://doi.org/10.6084/m9.figshare.12114129).
