# Supplementary material for: Culicidae evolutionary history focusing on the Culicinae subfamily based on mitochondrial phylogenomics
Source: Sci Rep. 2020 Nov 2;10:18823. doi: 10.1038/s41598-020-74883-3 (PMC7606482; doi:10.1038/s41598-020-74883-3)
Supplement: Supplementary file 1 — Supplementary Information. [file 41598_2020_74883_MOESM1_ESM.pdf]

Additional information for

Culicidae evolutionary history focusing on the Culicinae subfamily based on mitochondrial phylogenomics

Alexandre Freitas da Silva[1], Laís Ceschini Machado[1], Marcia Bicudo de Paula[2], Carla Júlia da Silva Pessoa[3] Vieira, Roberta Vieira de Morais Bronzoni[3], Maria Alice Varjal de Melo Santos[1], Gabriel Luz Wallau[1,*]

[1] Programa de Pós-Graduação em Biociências e Biotecnologia em Saúde; Departamento de Entomologia, Fundação Oswaldo Cruz - Instituto Aggeu Magalhães, Recife, 50670-420, Brasil, [2] Faculdade de Saúde Pública, Universidade de São Paulo, São Paulo, 01246-904, Brasil, [3] Universidade Federal de Mato Grosso, Sinop, 78550-728, Brasil.
*gabriel.wallau@cpqam.fiocruz.br

Supplementary table 1. Annotation of draft mitogenomes using MITOS.

Species	*nad2*	*COI*	*COII*	*atp8*	*atp6*	*COIII*	*nad3*	*nad5*	*nad4*	*nad4l*	*nad6*	*cytb*	*nad1*	*rrnL*	*rrnS*	*tRNA*	*PCG*	*rRNA*	Total of genes
Ad. squamipennis	1	1	1	-	-	1	1	1	**1**	-	-	1	1	**1**	1	16	9	2	27
Ae. alboannulatus	1	1	1	-	-	**1**	1	1	1	**1**	1	**1**	**1**	1	1	13	11	2	26
Ae. camptorhynchus	1	1	1	1	-	**1**	1	**1**	1	**1**	1	**1**	**1**	1	1	16	12	2	30
Ae. detritus	1	1	1	1	1	**1**	1	**1**	**1**	**1**	1	**1**	**1**	1	1	16	13	2	31
Ae. fluviatilis	1	1	1	1	1	1	-	**1**	1	**1**	1	**1**	1	1	-	4	12	1	17
Ae. polynesiensis	1	1	1	**1**	-	**1**	1	**1**	**1**	1	1	**1**	**1**	1	1	20	12	2	34
Ae. riversi	-	1	1	1	1	1	1	**1**	1	-	-	**1**	1	1	-	6	10	1	17
Ae. scapularis	1	**1**	1	1	**1**	1	-	**1**	1	-	1	**1**	1	-	1	13	11	1	25
Ae. taeniorhynchus	1	1	1	1	1	1	1	1	1	**1**	1	1	1	1	1	22	13	2	37
An. albimanus	1	1	1	**1**	-	**1**	1	**1**	**1**	**1**	1	**1**	**1**	1	1	20	12	2	34
An. aquasalis	1	1	1	1	-	**1**	-	1	1	-	-	**1**	1	1	**1**	3	9	2	14
An. freeborni	1	1	1	**1**	-	**1**	1	**1**	1	**1**	1	**1**	**1**	1	1	19	12	2	33
An. nuneztovari	1	1	1	1	-	**1**	1	**1**	1	1	1	**1**	1	1	1	9	12	2	23
An. quadriannulatus	1	1	1	**1**	-	**1**	1	**1**	**1**	**1**	1	**1**	**1**	1	1	20	12	2	34
Cq. albicosta	1	1	1	**1**	1	1	1	1	**1**	**1**	1	1	1	1	1	21	13	2	36
Cq. chrysonotum	**1**	1	1	-	1	1	1	1	1	1	1	**1**	1	1	1	20	12	2	34
Cq. hermanoi	**1**	1	1	1	1	1	1	**1**	1	-	-	1	-	1	1	14	10	2	26
Cq. juxtamansonia	1	1	1	-	1	1	-	1	1	-	-	1	-	1	1	8	8	2	18
Cq. venezuelensis	1	1	1	1	1	1	1	**1**	**1**	**1**	1	1	1	1	1	19	13	2	34
Cx. amazonensis	1	1	1	**1**	1	1	1	1	1	1	1	1	1	1	1	21	13	2	36
Cx. australicus	1	1	1	1	1	1	1	**1**	1	**1**	1	**1**	1	1	1	17	13	2	32
Cx. corniger	1	1	**1**	1	-	1	-	1	1	1	-	1	-	1	**1**	9	9	2	20
Cx. globocoxitus	1	1	1	1	-	**1**	1	**1**	1	**1**	1	**1**	**1**	1	1	17	12	2	31
Cx. hortensis	1	1	1	-	-	**1**	1	**1**	1	1	1	**1**	**1**	1	1	12	11	2	25
Cx. pipiens molestus	**1**	1	1	1	1	1	1	**1**	1	**1**	1	**1**	1	1	1	6	13	2	21
Cx. nigripalpus	1	1	1	1	1	1	1	1	**1**	**1**	1	1	1	1	1	22	13	2	37
Cx. tarsalis	1	1	1	1	-	**1**	1	**1**	**1**	**1**	1	**1**	**1**	1	1	19	12	2	33
Cx. torrentium	1	1	1	1	1	1	1	**1**	1	**1**	1	**1**	1	1	1	11	13	2	26
Ma. wilsoni	1	1	1	1	1	1	1	1	**1**	1	-	1	1	1	1	14	12	2	28
Ma.humeralis	-	1	**1**	-	1	-	1	**1**	-	-	-	1	1	1	1	5	7	2	14
Ma.titillans	**1**	1	**1**	-	1	1	1	1	**1**	-	1	1	1	1	1	14	11	2	27
Ps. cingulata	**1**	1	1	1	1	1	1	1	**1**	1	**1**	**1**	**1**	1	1	21	13	2	36
Ps.albipes	1	1	1	1	-	**1**	1	**1**	**1**	**1**	1	1	**1**	1	1	19	12	2	33
Tp. aranoides	1	1	-	-	-	**1**	-	1	-	1	-	**1**	1	1	1	-	7	2	9
Tr. digitatum	**1**	1	1	-	1	1	1	1	**1**	**1**	-	**1**	-	**1**	1	16	10	2	28
Tx. amboinensis	1	1	1	1	1	**1**	1	**1**	**1**	**1**	1	**1**	1	**1**	1	19	13	2	34
Ur. pulcherrima	1	**1**	1	-	1	1	1	1	1	1	1	**1**	1	1	1	18	12	2	32

Legend: Bold names represent sequenced mitogenomes. Disrupted or duplicated gene are represented by bold numbers. Unidentified gene are represented by -.

Supplementary table 2. Predicted TMRCA values from Bayesian inference analysis for studied species clades.

Node*	HPD 95%[1]	Median dating[1]	PP of analysis [1]	PP of analysis [2]	UFboot of analysis [2]	PP of analysis [3]	PP of analysis [4]	PP of analysis [5]	UFboot of analysis [5]	PP of analysis[6]	UFboot of analysis[7]
A	243.79-332.41	273.74	1	1	-	1	1	1	-	1	-
B	145.88-232.95	182.75	1	1	-	1	1	1	-	1	-
F	128.09-204.91	160.86	1	-	-	-	-	-	-	-	-
I	124.77-200.69	157.02	0.49	0.81	-	0.49	-	0.96	69	0.99	-
H	117.92-195.06	151.36	0.5	-	-	-	-	-	-	-	-
C	114.58-187.29	145.83	1	1	100	1	1	1	100	1	100
N	101.87-168.21	130.49	1	1	93	1	1	0.99	92	1	79
J	88.78-146.1	113.73	1	1	100	1	1	-	-	0.81	83
D	87.14-141.71	110.73	1	1	100	1	1	1	100	1	100
U	79.07-135.87	103.9	1	1	100	1	1	1	100	1	100
O	81.02-132.17	102.87	1	1	100	1	1	1	100	1	100
Q	74.02-121.02	94.04	1	1	100	1	1	1	100	1	-
K	66.37-115.22	88.32	1	1	100	1	1	1	100	1	100
L	66.31-109.73	85.29	1	1	100	1	1	1	100	1	100
S	53.53-88.54	68.94	1	-	100	1	1	-	-	-	-
P	45.84-84.94	63.25	1	1	100	1	1	1	100	1	100
T	43.26-73.19	56.37	0.92	-	65	0.95	-	-	-	-	-
R	24.82-45.09	33.9	1	1	100	1	1	1	100	1	100
V	16.73-28.91	22.09	1	1	100	1	-	-	100	0.85	89
Z	15.05-26.45	20.08	1	1	100	1	0.85	0.86	97	-	98
Y	9.82-17.37	13.15	1	1	-	1	1	-	-	0.96	99
X	7.78-16.85	11.85	1	1	100	1	1	1	100	1	99
M	3.13-8.92	5.71	1	1	100	1	1	1	100	1	100
E	2.35-4.42	3.27	1	1	100	1	1	1	100	1	100
G	1.43-2.81	2.04	1	1	100	1	1	1	-	1	-

The HPD95% values represent the confidence intervals for each node. PP represents the probability posterior. UFboot represents ultrafast bootstrapping. * Nodes present in Fig. 3 and Fig. 4. [1] Analysis based on partitioned PCG with codon partition (1st + 2nd and 3rd separately). [2] Analysis based on complete mitogenome sequences. [3] Analysis based on partitioned PCG without codon partition. [4] Analysis based on Partitioned PCG without 3rd codon positions. [5] Analysis based on concatenated predicted amino acid sequences. [6] Analysis based on partitioned predicted amino acid sequences. [7] Analysis based on concatenated PCG without 3rd codon position.

Supplementary table 3. Information of sequenced samples.

Taxon	Genus	Subgenus	Tribe	Nº of specimens	Location	Nº of reads (Mi)
Ad. squamipennis	Aedeomyia	Aedeomyia	Aedeomyiini	3	Aldeia-PE+REC[2]	3.9
Ae. scapularis	Aedes	Ochlerotatus	Aedini	7	Juazeiro-BA	7
Ae. taeniorhynchus	Aedes	Ochlerotatus	Aedini	10	São Luís -MA	11.3
Cq. hermanoi	Coquillettidia	Rhynchotaenia	Mansoniini	1	Aldeia-PE	1.8
Cq. chrysonotum	Coquillettidia	Rhynchotaenia	Mansoniini	14	PDI[1]	6.2
Cq. albicosta	Coquillettidia	Rhynchotaenia	Mansoniini	3	PDI[1]	2.4
Cq. juxtamansonia	Coquillettidia	Rhynchotaenia	Mansoniini	2	REC[2]	5.5
Cq. venezuelensis	Coquillettidia	Rhynchotaenia	Mansoniini	4	PDI[1]+REC[2]	4.3
Cx. amazonensis	Culex	Aedinus	Culicini	1	REC[2]	2.5
Cx. corniger	Culex	Phenacomyia	Culicini	5	Mato Grosso	3.9
Cx. nigripalpus	Culex	Culex	Culicini	6	PDI[1]	4.8
Ma. titillans	Mansonia	Mansonia	Mansoniini	3	REC[2]	6.6
Ma. wilsoni	Mansonia	Mansonia	Mansoniini	2	REC[2]	8.2
Ma. humeralis	Mansonia	Mansonia	Mansoniini	1	Aldeia-PE	2.2
Ps. cingulata	Psorophora	Grabhamia	Aedini	3	Mato Grosso	4.9
Tr. digitatum	Trichoprosopon	Trichoprosopon	Sabethini	1	Mato Grosso	7.6
Ur. pulcherrima	Uranotaenia	Uranotaenia	Uranotaeniini	1	REC[2]	1.1

PE refers to state of Pernambuco, Brazil. MA refers to state of Maranhão, Brazil. BA refers to state of Bahia, Brazil. Mi refers to million of reads. [1] Parque Estadual Dois Irmãos-PE . [2] Reserva Ecológica de Carnijó-PE

Supplementary table 4. NCBI accession number of mitochondrial genomes retrieved from NCBI.

Species	Genus	Subgenus	Tribe	Access	Length (bp)	References *
Aedes aegypti	Aedes	Stegomyia	Aedini	NC_010241.1	16,655	1
Aedes albopictus	Aedes	Stegomyia	Aedini	NC_006817.1	16,665	-
Aedes notoscriptus	Aedes	Finlaya	Aedini	KM676218.1	15,846	2
Aedes vigilax	Aedes	Ochlerotatus	Aedini	KP995260.1	15,877	3
Anopheles albitarsis	Anopheles	Nyssorhynchus	-	HQ335344.1	15,413	4
Anopheles bellator	Anopheles	Kerteszia	-	KU551287.1	15,668	5
Anopheles christyi	Anopheles	Cellia	-	NC_028214.1	14,967	6
Anopheles cruzii	Anopheles	Kerteszia	-	KJ701506.1	15,449	-
Anopheles culicifacies	Anopheles	Cellia	-	NC_027502.1	15,330	7
Anopheles darlingi	Anopheles	Nyssorhynchus	-	NC_014275.1	15,386	8
Anopheles deaneorum	Anopheles	Nyssorhynchus	-	HQ335347.1	15,424	4
Anopheles dirus	Anopheles	Cellia	-	JX219731.1	15,404	9
Anopheles epiroticus	Anopheles	Cellia	-	NC_028217.1	15,379	6
Anopheles farauti	Anopheles	Cellia	-	NC_020770.1	15,412	9
Anopheles funestus	Anopheles	Cellia	-	NC_008070.1	15,354	10
Anopheles gambiae	Anopheles	Cellia	-	NC_002084.1	15,363	11
Anopheles homunculus	Anopheles	Kerteszia	-	KU551283.1	15,739	5
Anopheles janconnae	Anopheles	Nyssorhynchus	-	HQ335348.1	15,425	4
Anopheles koliensis	Anopheles	Cellia	-	JX219743.1	15,412	9
Anopheles melas	Anopheles	Cellia	-	NC_028219.1	15,366	6
Anopheles merus	Anopheles	Cellia	-	NC_028220.1	15,365	6
Anopheles punctulatus	Anopheles	Cellia	-	NC_028222.1	15,322	6
Anopheles quadrimaculatus	Anopheles	Anopheles	-	NC_000875.1	15,455	12
Anopheles sinensis	Anopheles	Anopheles	-	NC_028016.1	15,076	-
Anopheles stephensi	Anopheles	Cellia	-	NC_028223.1	15,387	6
Bironella hollandi	Bironella	Brugella	-	NC_037796.1	15,772	13
Chagasia sp.	Chagasia	-	-	MF381717.1	15,717	13
Culex bidens	Culex	Culex	Culicini	NC_037809.1	15,583	13
Culex gelidus	Culex	Culex	Culicini	KX753344.1	15,600	14
Culex bilineatus	Culex	Culex	Culicini	NC_037819.1	15,599	13
Culex brami	Culex	Culex	Culicini	NC_037828.1	15,586	13
Culex camposi	Culex	Culex	Culicini	NC_036008.1	15,570	15

Culex coronator	Culex	Culex	Culicini	NC_036006.1	15,576	15
Culex declarator	Culex	Culex	Culicini	NC_037822.1	15,575	13
Culex dolosus	Culex	Culex	Culicini	MF381620.1	15,851	13
Culex chidesteri	Culex	Culex	Culicini	NC_037826.1	16,052	13
Culex lygrus	Culex	Culex	Culicini	MF381718.1	15,572	13
Culex mollis	Culex	Culex	Culicini	MF381705.1	15,576	13
Culex pipiens pallens	Culex	Culex	Culicini	KT851543.1	15,617	16
Culex pipiens pipiens	Culex	Culex	Culicini	NC_015079.1	14,856	-
Culex quinquefasciatus	Culex	Culex	Culicini	NC_014574.1	15,587	1
Culex surinamensis	Culex	Culex	Culicini	MF381615.1	15,568	13
Culex tritaeniorhynchus	Culex	Culex	Culicini	KT851544.1	14,844	16
Culex usquatissimus	Culex	Culex	Culicini	NC_036007.1	15,574	15
Culex usquatus	Culex	Culex	Culicini	NC_036005.1	15,573	15
Drosophila melanogaster	Drosophila	Drosophilinae	Drosophilini	U37541.1	19,517	17
Haemagogus janthinomys	Haemagogus	Haemagogus	Aedini	NC_028025.1	15,698	18
Sabethes belisarioi	Sabethes	Sabethes	Sabethini	MF957171.1	15,911	19
Sabethes chloropterus	Sabethes	Sabethoides	Sabethini	NC_037499.1	15,609	19
Sabethes glaucodaemon	Sabethes	Sabethoides	Sabethini	NC_037500.1	15,620	19

* References listed at the end of the document.

Supplementary table 5. Information about retrieved raw sequencing datasets from NCBI SRA database.

**Species**	**Genus**	**Subgenus**	**Tribe**	**SRA accession number**	**Original study**
Ae. alboannulatus	Aedes	Finlaya	Aedini	SRR5665565	RNA-Seq (Species virome)
Ae. camptorhynchus	Aedes	Ochlerotatus	Aedini	SRR5665568	RNA-Seq (Species virome)
Ae. detritus	Aedes	Ochlerotatus	Aedini	SRR6489869	RNA-Seq (Differential gene expression)
Ae. fluviatilis	Aedes	Ochlerotatus	Aedini	SRR3574348	RNA-Seq (Differential gene expression)
Ae. polynesiensis	Aedes	Stegomyia	Aedini	ERR1706659	RNA-Seq (Comparative transcriptomes)
Ae. riversi	Aedes	Stegomyia	Aedini	ERR1706661	RNA-Seq (Comparative transcriptomes)
An. albimanus	Anopheles	Nyssorhynchus	-	SRR314655	Genome sequencing
An. aquasalis	Anopheles	Nyssorhynchus	-	SRR927456	RNA-Seq (Transcriptome)
An. freeborni	Anopheles	Anopheles	-	SRR908289	RNA-Seq (Saliva transcriptome)
An. nuneztovari	Anopheles	Nyssorhynchus	-	SRR6471062	RNA-Seq (Saliva transcriptome)
An. quadriannulatus	Anopheles	Cellia	-	SRR529986	Genome sequencing
Cx. australicus	Culex	Culex	Culicini	SRR5665566	RNA-Seq (Species virome)
Cx. globocoxitus	Culex	Culex	Culicini	SRR5665567	RNA-Seq (Species virome)
Cx. hortensis	Culex	Maillotia	Culicini	SRR1324883	RNA-Seq (Comparative transcriptomes)
Cx. pipiens molestus	Culex	Culex	Culicini	SRR1462325	RNA-Seq (Comparative transcriptomes)
Cx. tarsalis	Culex	Culex	Culicini	SRR5149179	RNA-Seq (Transcriptome)
Cx. torrentium	Culex	Culex	Culicini	SRR1324895	RNA-Seq

					(Comparative transcriptomes)
Ps.albipes	Psorophora	Janthinossoma	Aedini	SRR908278	RNA-Seq (Saliva transcriptome)
Tp. aranoides	Tripteroides	Rachionotomyia	Sabethini	SRR6155938	RNA-Seq (Transcriptome)
Tx. amboinensis	Toxorhynchites	Toxorhynchites	Toxorhynchitini	SRR2061845	RNA-Seq (Transcriptome)

Supplementary table 6. Calibration points used in BEAST 1.8.4

Clade	Represented taxa	Minimum bound	Maximum bound	References
Diptera	*Culicidae* and *D. melanogaster*	240	241	KRZEMIŃSKI, KRZEMIŃSKA & PAPIER, 1994 [20]
Culicidae	*Culicinae* e *Anophelinae*	90	99	BORKENT & GRIMALDI, 2004 [21]
Culicinae	*Culicinae*	76.5	79	POINAR, ZAVORTINK., PIKE & JOHNSTON, 2000 [22]
Anophelinae	*Anophelinae*	15	34	ZAVORTINK & POINAR, 2000 [23]

Supplementary table 7. Evolutionary models used in BEAST analysis.

Partition	Best nucleotide model [1]	Best protein model [2]
Nucleotide mitogenome alignment	GTR+G+I	-
Concatenated protein alignment	-	mtREV+G+I
Atp6	GTR+G+I	mtREV+G+I
Atp8	GTR+G	JTT+G+I
COIII	GTR+G+I	mtREV+G+I
COII	GTR+G+I	mtREV+G
COI	GTR+G+I	JTT+G+I
CytB	GTR +G+I	mtREV+G+I
Nad1	GTR +G+I	JTT+G+I
Nad2	GTR +G+I	JTT+G
Nad3	GTR +G+I	mtREV+G+I
Nad4L	GTR +G+I	mtREV+G
Nad4	GTR +G+I	JTT+G+I
Nad5	GTR +G+I	JTT+G
Nad6	GTR +G+I	mtREV+G
rrnL	GTR +G+I	-
rrnS	GTR +G+I	-

[1]Best model as suggested by SMS.[2] More likely model suggested by Prottest and present in BEAST 1.8.4.

Supplementary file 1. Test of substitution saturation of mitochondrial PCG.
Analysis performed on all sites.
Testing whether the observed Iss is significantly lower than Iss.c.
IssSym is Iss.c assuming a symmetrical topology.
IssAsym is Iss.c assuming an asymmetrical topology.
Note: two-tailed tests are used.

COI 1st+2nd codon positions:

NumOTU	Iss	Iss.cSym	T	DF	P	Iss.cAsym	T	DF	P
4	0,119	0,821	72,061	1023	0,0000	0,789	68,777	1023	0,0000
8	0,120	0,789	64,934	1023	0,0000	0,684	54,715	1023	0,0000
16	0,124	0,773	64,019	1023	0,0000	0,576	44,550	1023	0,0000
32	0,147	0,750	59,214	1023	0,0000	0,445	29,334	1023	0,0000

COI 3rd codon positions:

NumOTU	Iss	Iss.cSym	T	DF	P	Iss.cAsym	T	DF	P
4	0,537	0,796	11,883	510	0,0000	0,762	10,330	510	0,0000
8	0,580	0,753	7,928	510	0,0000	0,641	2,812	510	0,0051
16	0,600	0,722	5,837	510	0,0000	0,513	4,163	510	0,0000
32	0,608	0,703	4,751	510	0,0000	0,378	11,490	510	0,0000

COII 1st+2nd codon positions:

NumOTU	Iss	Iss.cSym	T	DF	P	Iss.cAsym	T	DF	P
4	0,258	0,793	31,684	456	0,0000	0,759	29,701	456	0,0000
8	0,224	0,747	30,309	456	0,0000	0,636	23,846	456	0,0000
16	0,274	0,713	25,787	456	0,0000	0,504	13,505	456	0,0000
32	0,285	0,697	25,475	456	0,0000	0,371	5,276	456	0,0000

COII 3rd codon positions:

NumOTU	Iss	Iss.cSym	T	DF	P	Iss.cAsym	T	DF	P
4	0,630	0,777	4,490	227	0,0000	0,762	4,033	227	0,0001
8	0,673	0,732	1,878	227	0,0617	0,631	1,319	227	0,1885
16	0,718	0,654	2,087	227	0,0380	0,459	8,498	227	0,0000
32	0,750	0,685	2,250	227	0,0254	0,363	13,387	227	0,0000

COIII 1st+2nd codon positions:

NumOTU	Iss	Iss.cSym	T	DF	P	Iss.cAsym	T	DF	P
4	0,230	0,797	34,999	525	0,0000	0,764	32,946	525	0,0000
8	0,236	0,754	32,263	525	0,0000	0,643	25,328	525	0,0000
16	0,261	0,725	29,985	525	0,0000	0,515	16,431	525	0,0000
32	0,281	0,705	29,101	525	0,0000	0,378	6,659	525	0,0000

COIII 3rd codon positions:

NumOTU	Iss	Iss.cSym	T	DF	P	Iss.cAsym	T	DF	P
4	0,651	0,778	4,004	262	0,0001	0,758	3,356	262	0,0009

NumOTU	Iss	Iss.cSym	T	DF	P	Iss.cAsym	T	DF	P
8	0,716	0,732	0,487	262	0,6265	0,627	2,761	262	0,0062
16	0,748	0,666	2,525	262	0,0121	0,465	8,773	262	0,0000
32	0,796	0,682	3,501	262	0,0005	0,356	13,527	262	0,0000

ND1 1st+2nd codon positions:

NumOTU	Iss	Iss.cSym	T	DF	P	Iss.cAsym	T	DF	P
4	0,232	0,803	41,973	627	0,0000	0,772	39,681	627	0,0000
8	0,270	0,763	31,688	627	0,0000	0,653	24,621	627	0,0000
16	0,257	0,741	33,136	627	0,0000	0,530	18,739	627	0,0000
32	0,307	0,715	27,312	627	0,0000	0,388	5,436	627	0,0000

ND1 3rd codon positions:

NumOTU	Iss	Iss.cSym	T	DF	P	Iss.cAsym	T	DF	P
4	0,616	0,782	5,860	313	0,0000	0,756	4,938	313	0,0000
8	0,773	0,735	1,302	313	0,1939	0,626	5,017	313	0,0000
16	0,787	0,682	3,693	313	0,0003	0,475	10,927	313	0,0000
32	0,776	0,683	3,322	313	0,0010	0,354	15,165	313	0,0000

ND2 1st+2nd codon positions:

NumOTU	Iss	Iss.cSym	T	DF	P	Iss.cAsym	T	DF	P
4	0,462	0,806	19,700	683	0,0000	0,775	17,927	683	0,0000
8	0,505	0,768	14,386	683	0,0000	0,658	8,401	683	0,0000
16	0,505	0,747	13,360	683	0,0000	0,538	1,812	683	0,0704
32	0,598	0,721	6,691	683	0,0000	0,396	10,982	683	0,0000

ND2 3rd codon positions:

NumOTU	Iss	Iss.cSym	T	DF	P	Iss.cAsym	T	DF	P
4	0,778	0,784	0,226	341	0,8212	0,756	0,810	341	0,4185
8	0,828	0,737	3,275	341	0,0012	0,627	7,222	341	0,0000
16	1,031	0,689	12,631	341	0,0000	0,481	20,299	341	0,0000
32	1,037	0,685	13,325	341	0,0000	0,356	25,823	341	0,0000

ND3 1st+2nd codon positions:

NumOTU	Iss	Iss.cSym	T	DF	P	Iss.cAsym	T	DF	P
4	0,367	0,777	16,360	233	0,0000	0,761	15,719	233	0,0000
8	0,332	0,732	15,880	233	0,0000	0,630	11,819	233	0,0000
16	0,379	0,656	10,371	233	0,0000	0,460	3,005	233	0,0029
32	0,415	0,684	10,433	233	0,0000	0,362	2,066	233	0,0400

ND3 3rd codon positions:

NumOTU	Iss	Iss.cSym	T	DF	P	Iss.cAsym	T	DF	P
4	0,669	0,793	2,773	116	0,0065	0,821	3,396	116	0,0009
8	0,770	0,773	0,069	116	0,9452	0,707	1,383	116	0,1694
16	0,826	0,599	4,990	116	0,0000	0,469	7,845	116	0,0000
32	0,950	0,763	4,278	116	0,0000	0,512	10,022	116	0,0000

ND4L 1st+2nd codon positions:

NumOTU	Iss	Iss.cSym	T	DF	P	Iss.cAsym	T	DF	P
4	0,330	0,780	17,564	157	0,0000	0,784	17,747	157	0,0000
8	0,334	0,745	14,305	157	0,0000	0,658	11,299	157	0,0000
16	0,349	0,623	10,092	157	0,0000	0,454	3,879	157	0,0002
32	0,432	0,712	10,365	157	0,0000	0,417	0,545	157	0,5863

ND4L 3rd codon positions:

NumOTU	Iss	Iss.cSym	T	DF	P	Iss.cAsym	T	DF	P
4	0,659	0,833	3,248	78	0,0017	0,903	4,560	78	0,0000
8	0,707	0,848	2,665	78	0,0093	0,823	2,189	78	0,0316
16	0,793	0,573	4,465	78	0,0000	0,524	5,454	78	0,0000
32	0,839	0,891	1,139	78	0,2580	0,739	2,217	78	0,0296

ND4 1st+2nd codon positions:

NumOTU	Iss	Iss.cSym	T	DF	P	Iss.cAsym	T	DF	P
4	0,344	0,816	31,298	895	0,0000	0,785	29,198	895	0,0000
8	0,357	0,782	24,785	895	0,0000	0,675	18,555	895	0,0000
16	0,402	0,765	18,447	895	0,0000	0,563	8,178	895	0,0000
32	0,407	0,740	15,626	895	0,0000	0,428	0,971	895	0,3318

ND4 3rd codon positions:

NumOTU	Iss	Iss.cSym	T	DF	P	Iss.cAsym	T	DF	P
4	0,682	0,792	4,404	447	0,0000	0,759	3,068	447	0,0023
8	0,750	0,747	0,111	447	0,9118	0,635	4,355	447	0,0000
16	0,811	0,711	3,659	447	0,0003	0,502	11,350	447	0,0000
32	0,834	0,696	4,726	447	0,0000	0,369	15,958	447	0,0000

ND5 1st+2nd codon positions:

NumOTU	Iss	Iss.cSym	T	DF	P	Iss.cAsym	T	DF	P
4	0,390	0,826	28,458	1161	0,0000	0,794	26,372	1161	0,0000
8	0,375	0,796	23,303	1161	0,0000	0,692	17,559	1161	0,0000
16	0,413	0,780	17,244	1161	0,0000	0,587	8,194	1161	0,0000
32	0,473	0,758	11,503	1161	0,0000	0,461	0,452	1161	0,6514

ND5 3rd codon positions:

NumOTU	Iss	Iss.cSym	T	DF	P	Iss.cAsym	T	DF	P
4	0,723	0,801	3,337	580	0,0009	0,769	1,964	580	0,0501
8	0,823	0,759	2,356	580	0,0188	0,648	6,451	580	0,0000
16	0,885	0,734	4,978	580	0,0000	0,524	11,951	580	0,0000
32	0,973	0,710	7,593	580	0,0000	0,382	17,075	580	0,0000

ND6 1st+2nd codon positions:

NumOTU	Iss	Iss.cSym	T	DF	P	Iss.cAsym	T	DF	P

NumOTU	Iss	Iss.cSym	T	DF	P	Iss.cAsym	T	DF	P
4	0,463	0,781	12,219	307	0,0000	0,756	11,237	307	0,0000
8	0,528	0,734	7,426	307	111,3362	0,626	3,521	307	0,0005
16	0,564	0,680	4,021	307728760384		0,474	3,118	307	0,0020
32	0,632	0,683	1,713	307	0,0877	0,354	9,373	307	0,0002

ND6 3rd codon positions:

NumOTU	Iss	Iss.cSym	T	DF	P	Iss.cAsym	T	DF	P
4	0,847	0,781	1,620	152	0,1073	0,788	1,454	152	0,1479
8	0,986	0,747	5,532	15213484792,		0,662	7,495	152 510,3412	
16	1,049	0,620	10,045	152	0,0002	0,455	13,912	152	0,0000
32	1,118	0,716	9,303	152	0,0014	0,425	16,021	152	0,0000

Interpretation of results:

	Significant Difference	
	Yes	No
Iss < Iss.c	Little saturation	Substantial saturation

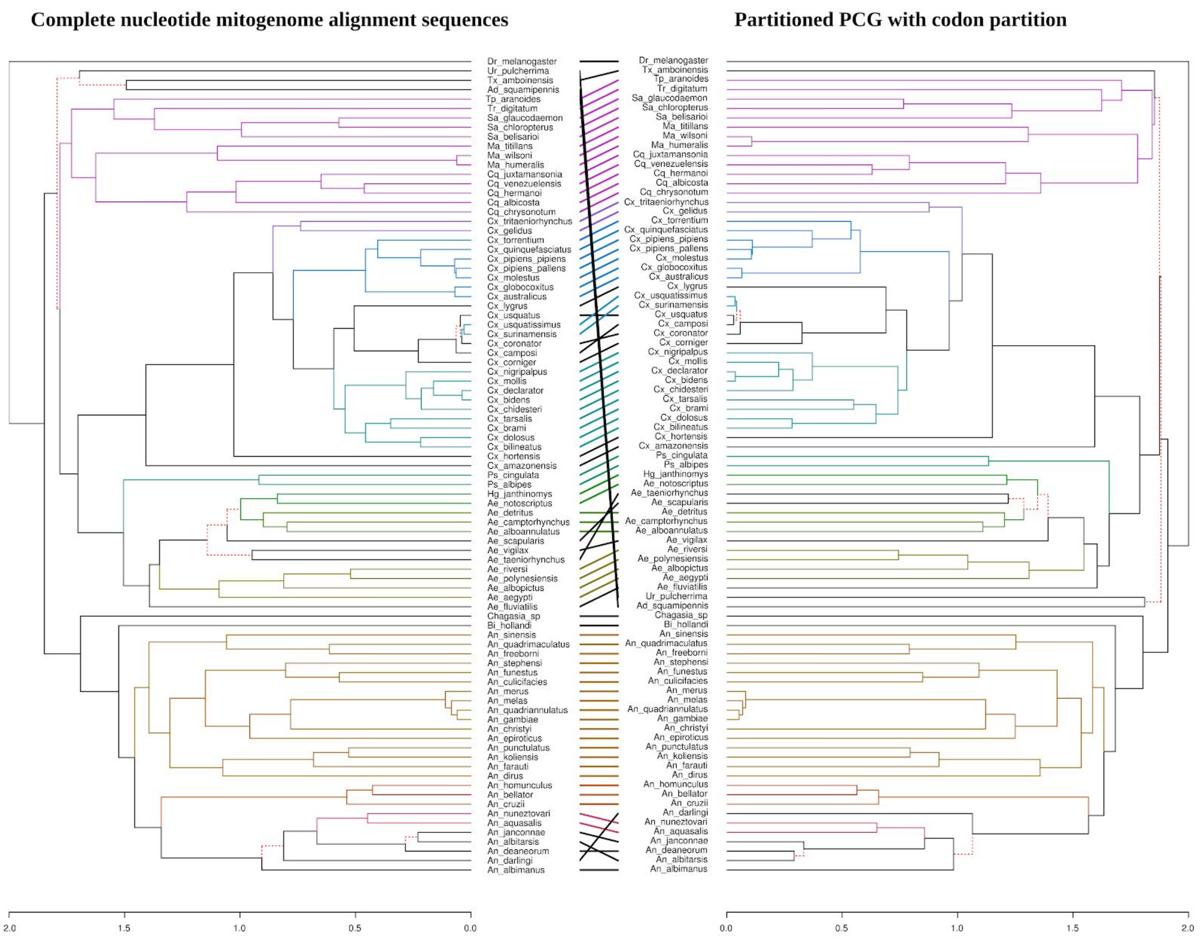

Supplementary figure 1. Comparative dendrogram topologies of trees obtained from different approaches using complete nucleotide mitogenomes and PCG with codon partition in the BEAST analysis. Dashed red lines show the branches that were different between the approaches used.

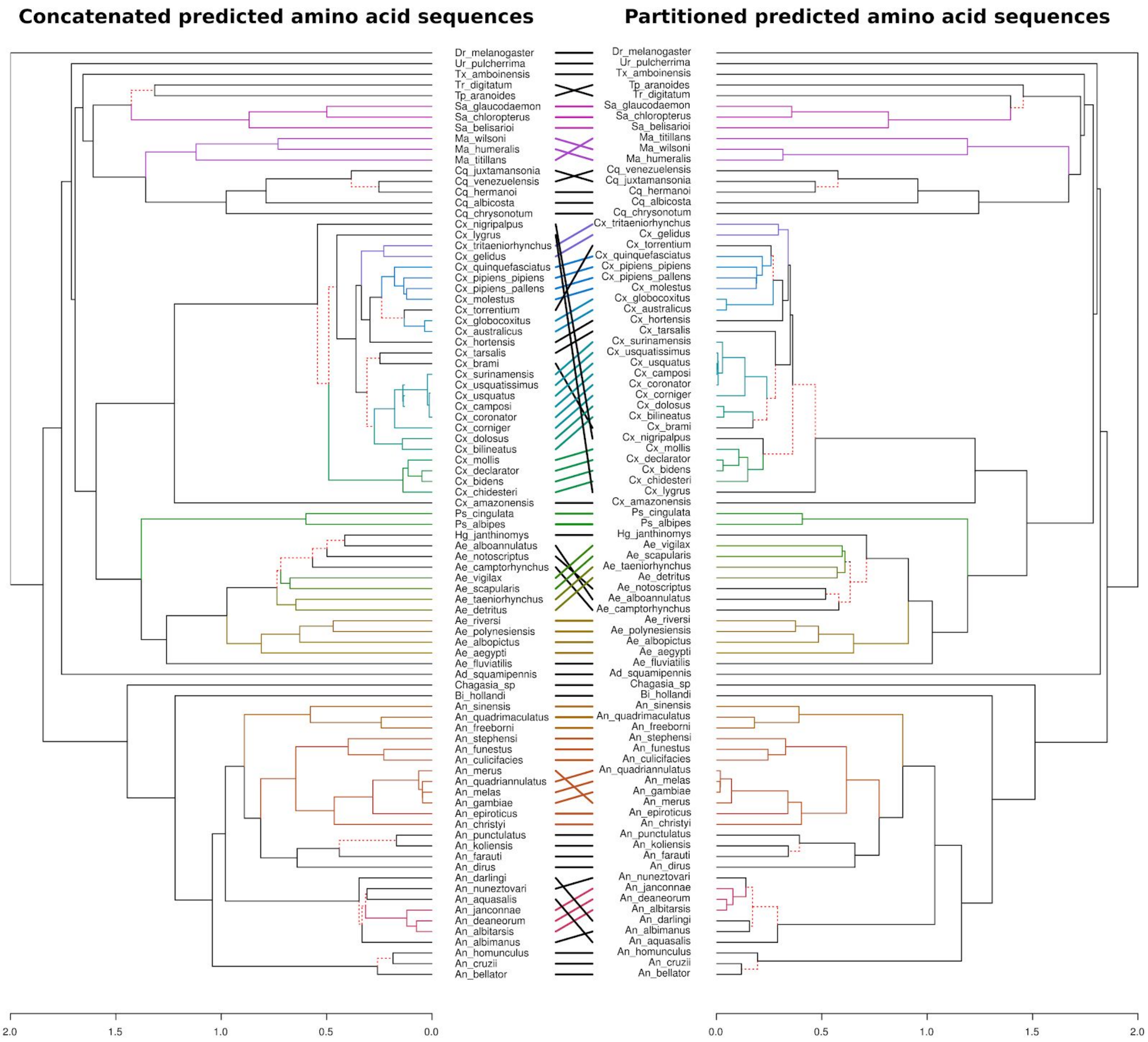

Supplementary figure 2. Comparative dendrogram topologies of trees obtained from different approaches using concatenated predicted amino acid sequences and partitioned predicted amino acid sequences in the BEAST analysis. Dashed red lines show the branches that were different between the approaches used.

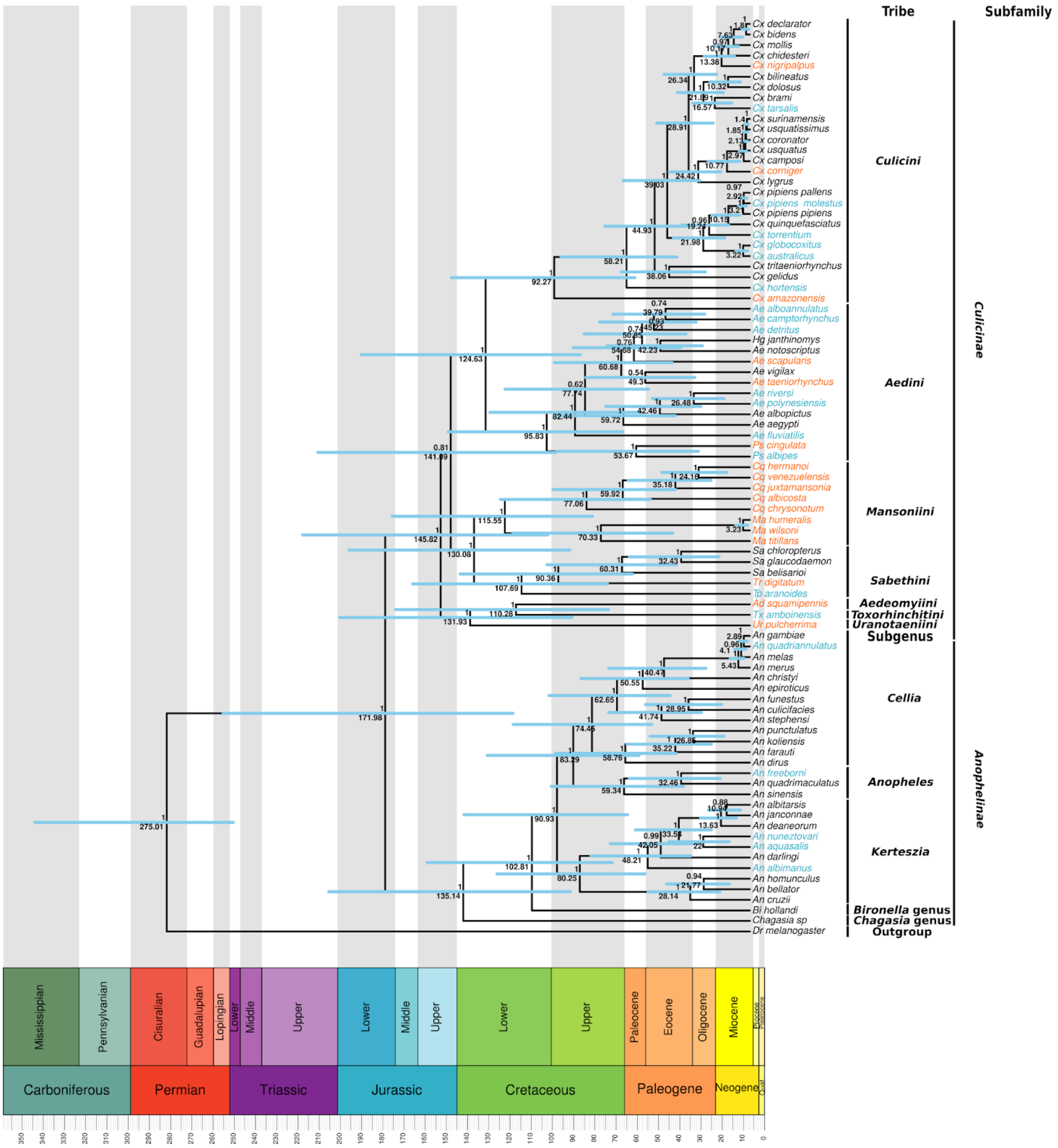

Supplementary figure 3. Evolutionary timescale of the Culicidae family based on BEAST analysis using complete mitogenome sequences. Blue bars represent the HPD95%. The numbers above and below the bars show the posterior probability and the predicted median dating respectively for each node. Light blue tip names represent mitogenomes characterized from SRA data. Orange tip names represent sequenced mitogenomes from this study.

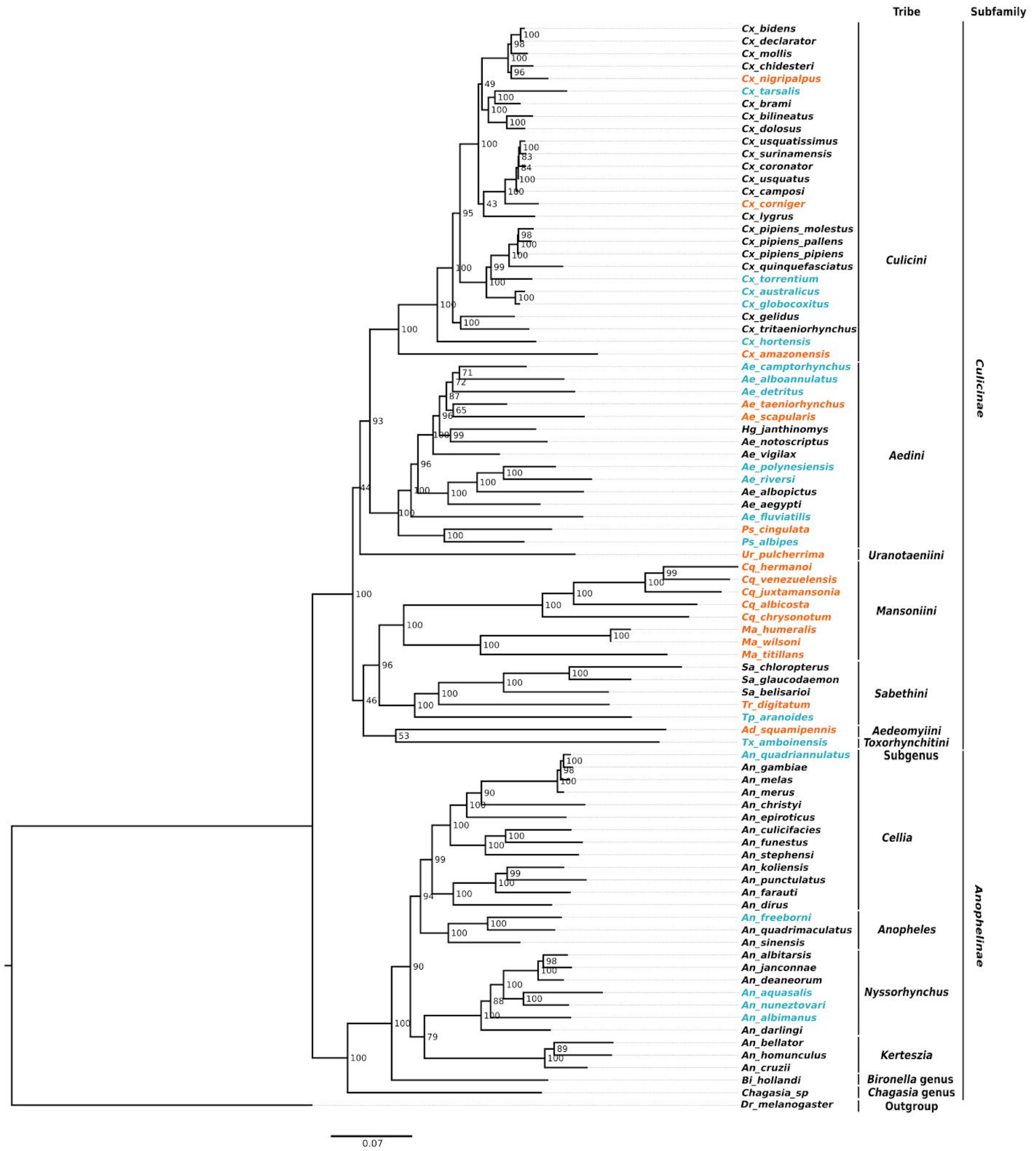

Supplementary figure 4. Maximum likelihood phylogenetic tree reconstructed with IQ-TREE version 1.6.12 based on complete mitogenome sequences using the GTR+F+I+G4 evolutionary model and the ultrafast bootstrapping with 1000 replicates. Light blue tip names represent mitogenomes characterized from SRA data. Orange tip names represent sequenced mitogenomes from this study.

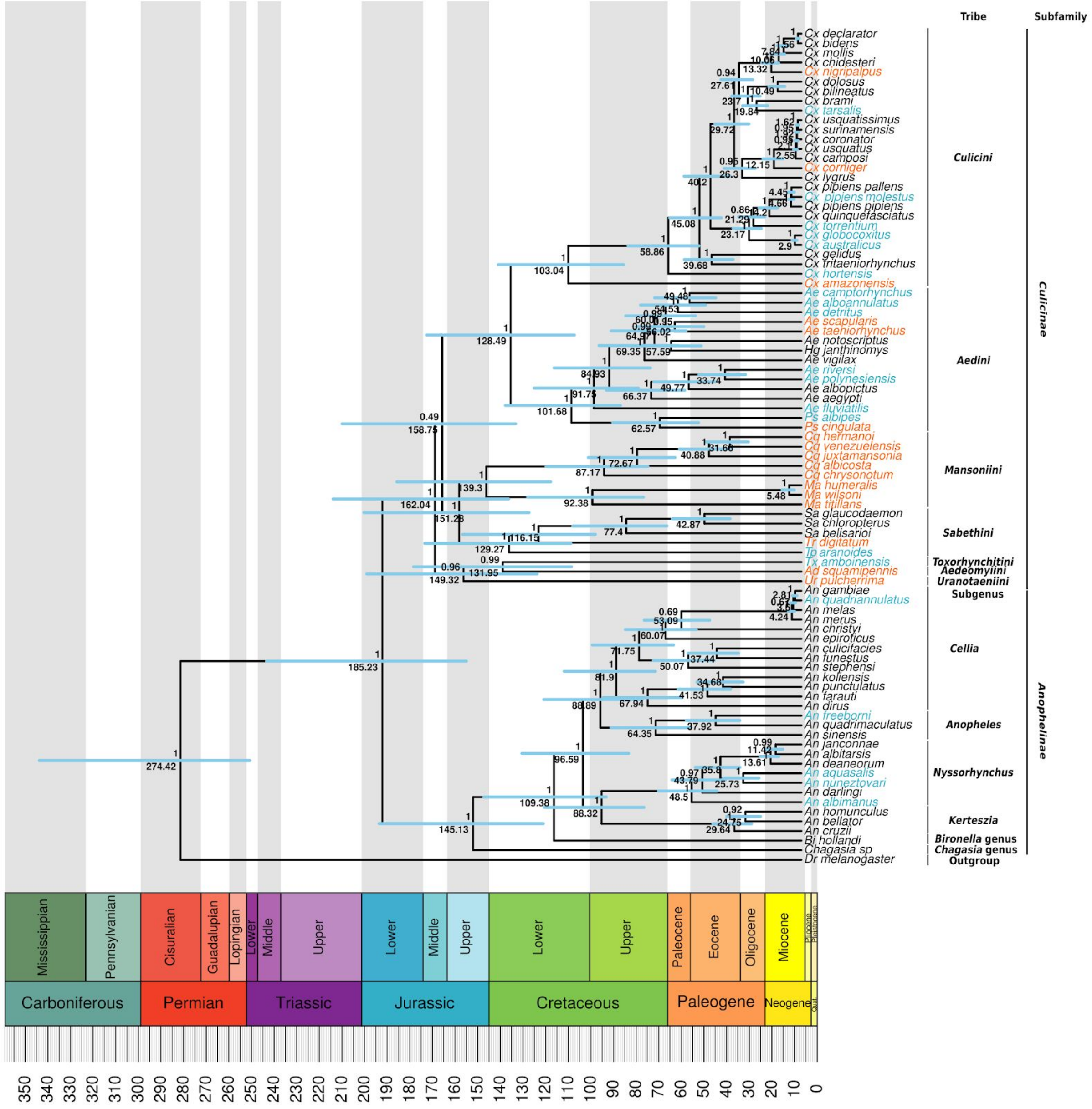

Supplementary figure 5. Evolutionary timescale of Culicidae family based on BEAST analysis using a partitioned scheme of PCG without codon partition. Blue bars represent the HPD95%. The numbers above and below the bars show the posterior probability and the predicted median dating respectively for each node. Light blue tip names represent mitogenomes characterized from SRA data. Orange tip names represent sequenced mitogenomes from this study.

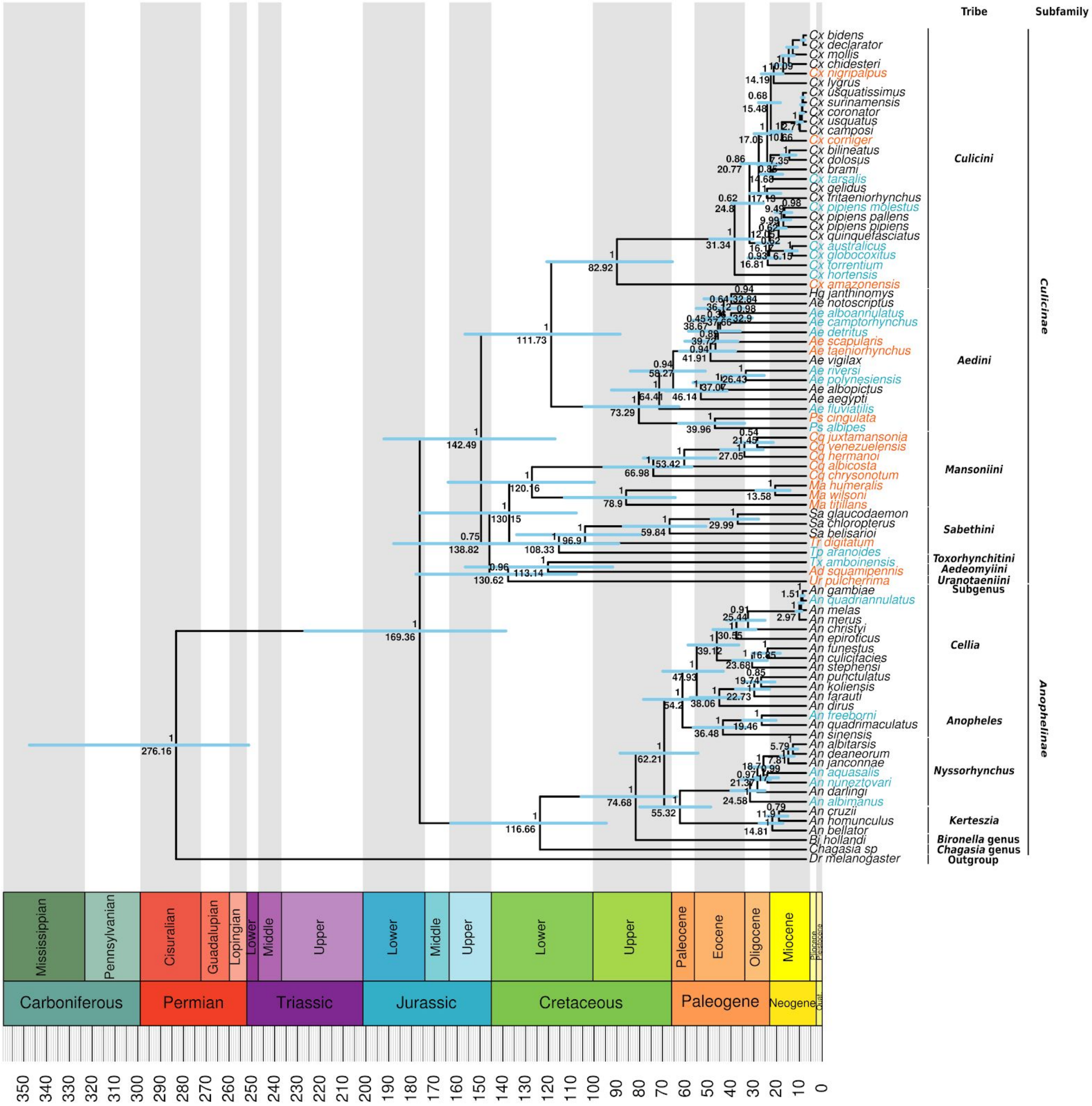

Supplementary figure 6. Evolutionary timescale of Culicidae family based on BEAST analysis using a partitioned scheme of PCG without 3rd codon positions. Blue bars represent the HPD95%. The numbers above and below the bars show the posterior probability and the predicted median dating respectively for each node. Light blue tip names represent mitogenomes characterized from SRA data. Orange tip names represent sequenced mitogenomes from this study.

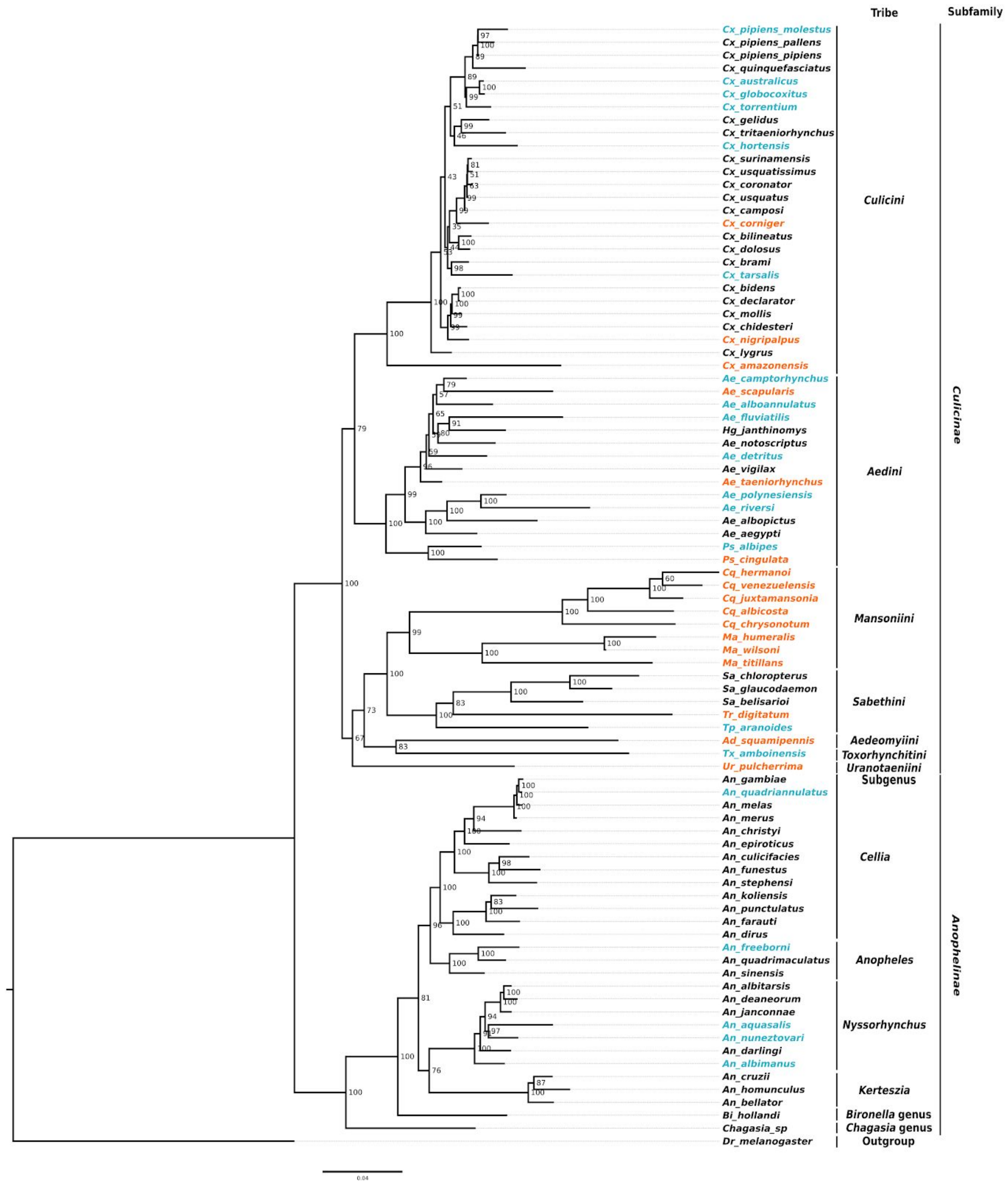

Supplementary figure 7. Maximum likelihood phylogenetic tree reconstructed with IQ-TREE version 1.6.12 based on concatenated PCG without 3rd codon positions using the GTR+F+I+G4 evolutionary model and the ultrafast bootstrapping with 1000 replicates. Light blue tip names represent mitogenomes characterized from SRA data. Orange tip names represent sequenced mitogenomes from this study.

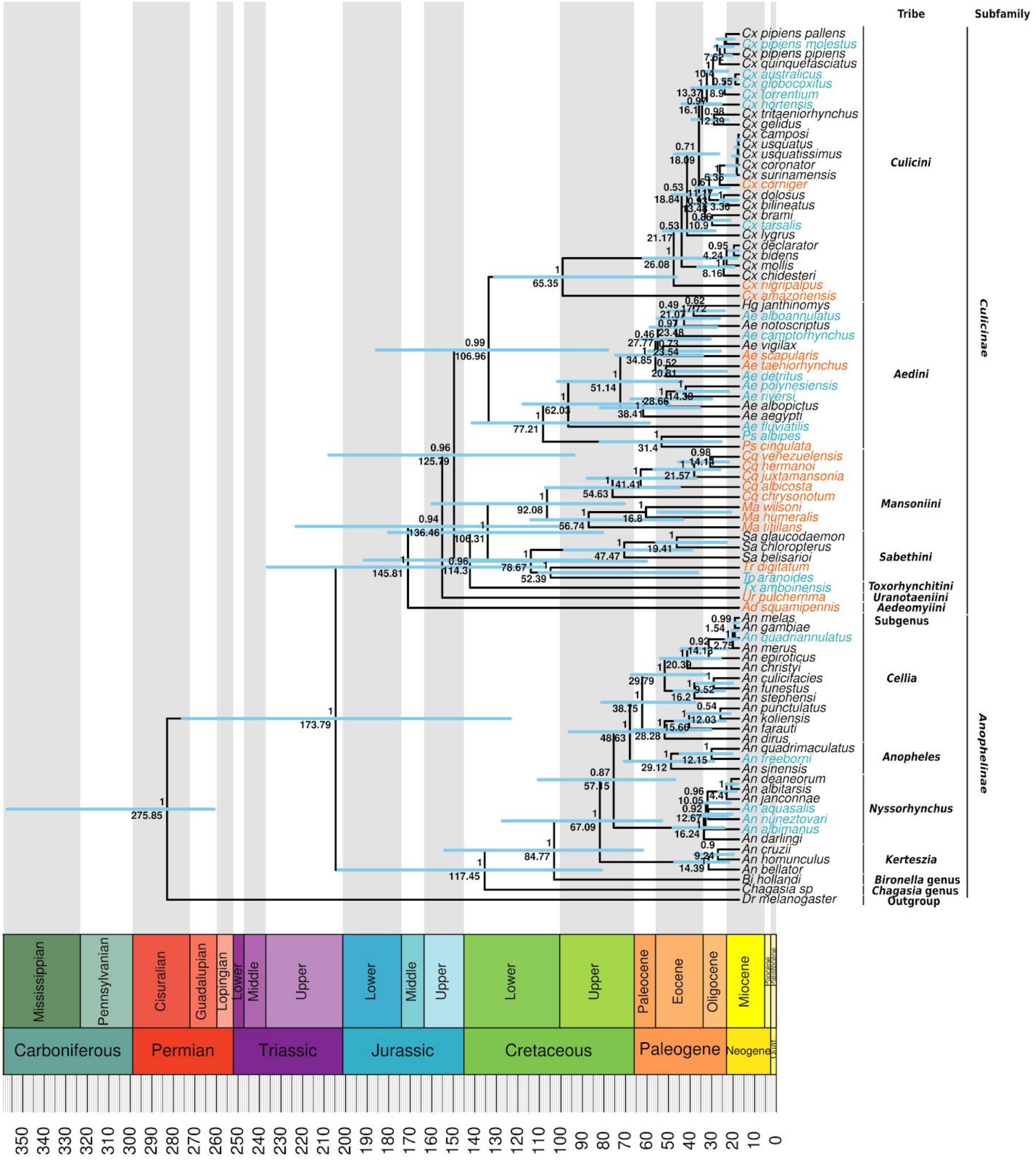

Supplementary figure 8. Evolutionary timescale of Culicidae family based on BEAST analysis using a concatenated predicted amino acid sequence. Blue bars represent the HPD95%. The numbers above and below the bars show the posterior probability and the predicted median dating respectively for each node. Light blue tip names represent mitogenomes characterized from SRA data. Orange tip names represent sequenced mitogenomes from this study.

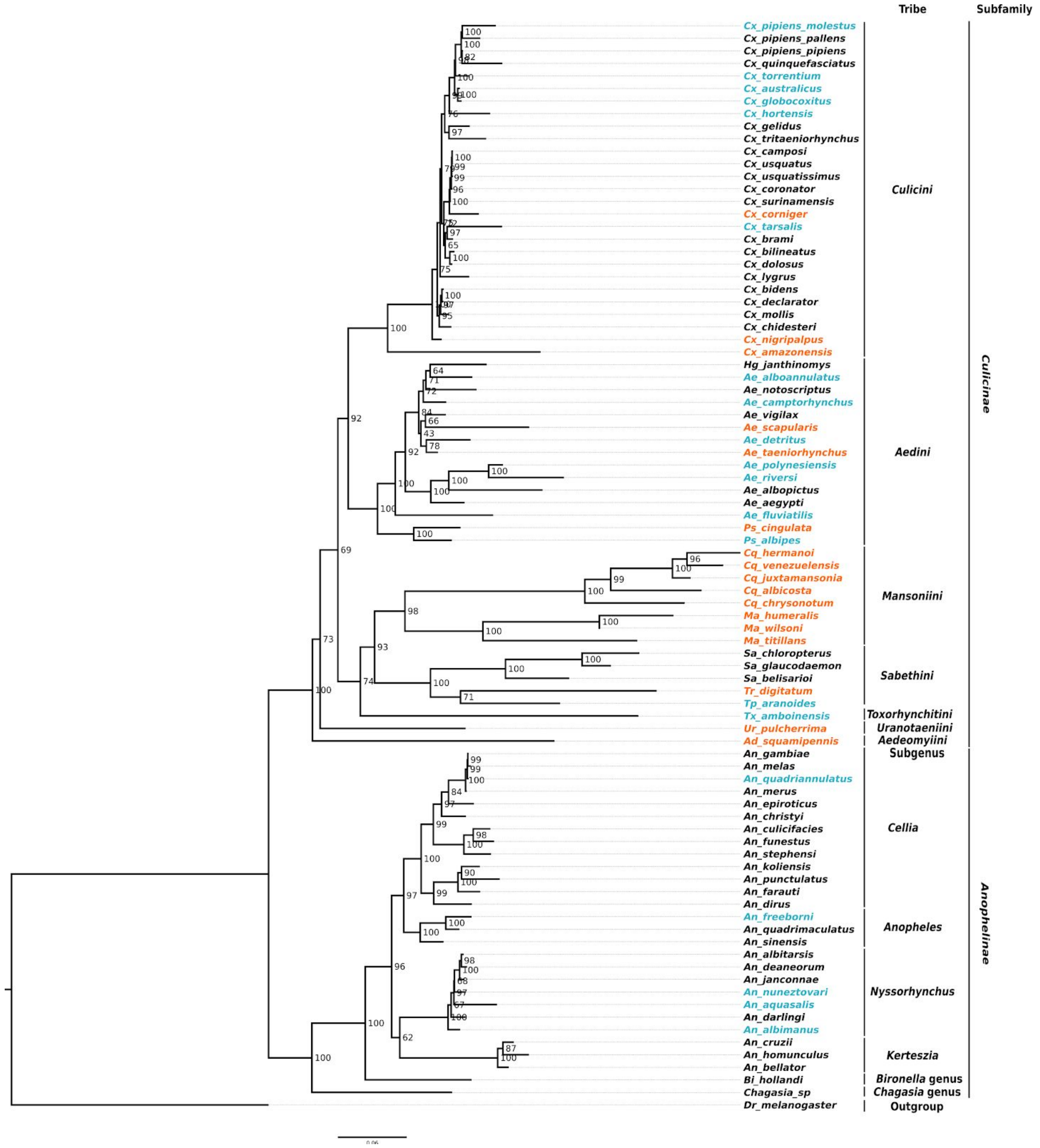

Supplementary figure 9. Phylogenetic tree of Maximum likelihood reconstructed with IQ-TREE version 1.6.12 based on concatenated predicted amino acid sequences using the mtInv+I+G4 evolutionary model and the ultrafast bootstrapping with 1000 replicates. Light blue tip names represent mitogenomes characterized from SRA data. Orange tip names represent sequenced mitogenomes from this study.

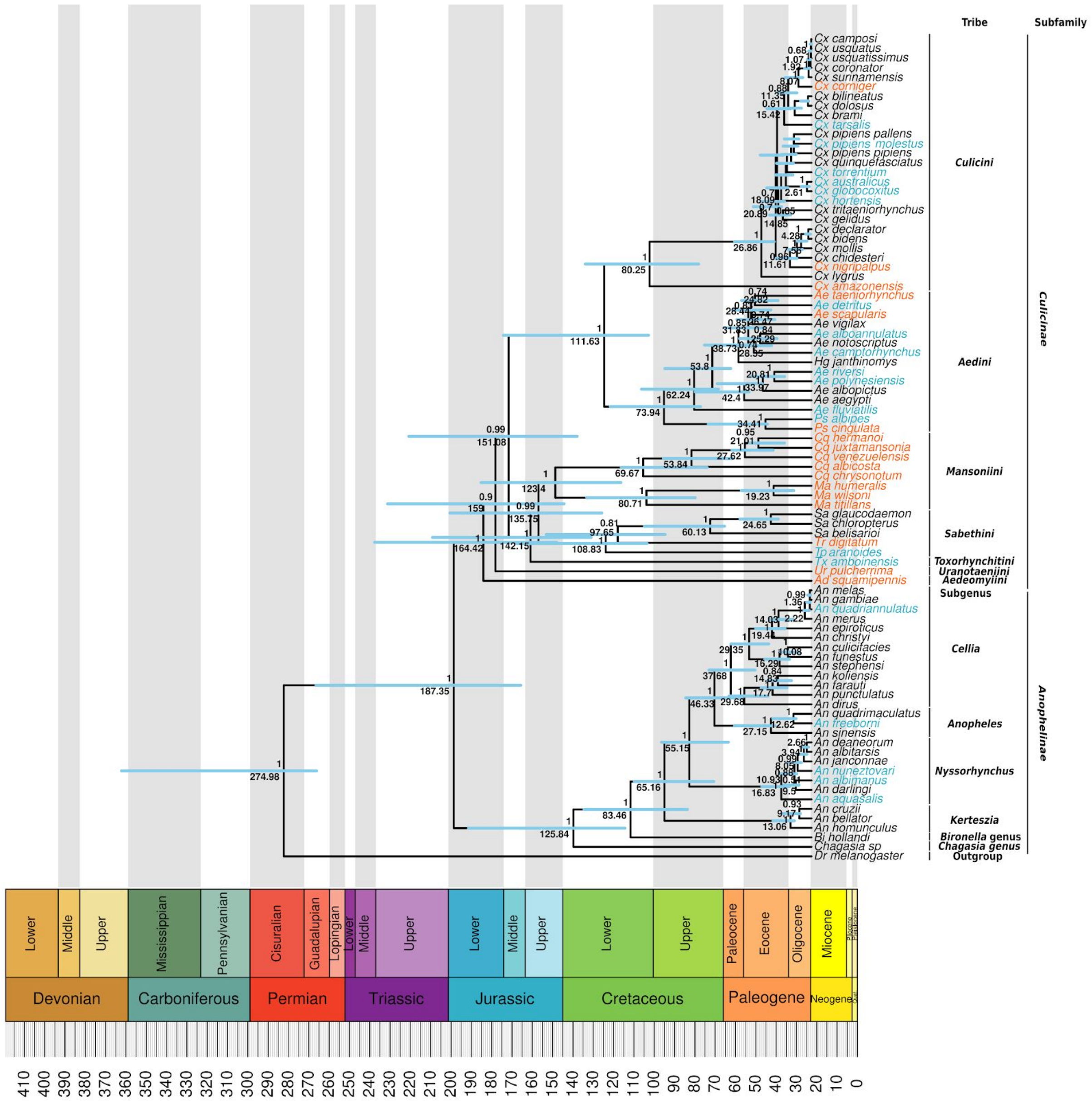

Supplementary figure 10. Evolutionary timescale of Culicidae family based on BEAST analysis using a partitioned scheme of predicted amino acid sequences. Blue bars represent the HPD95%. The numbers above and below the bars show the posterior probability and the predicted median dating respectively for each node. Light blue tip names represent mitogenomes characterized from SRA data. Orange tip names represent sequenced mitogenomes from this study.

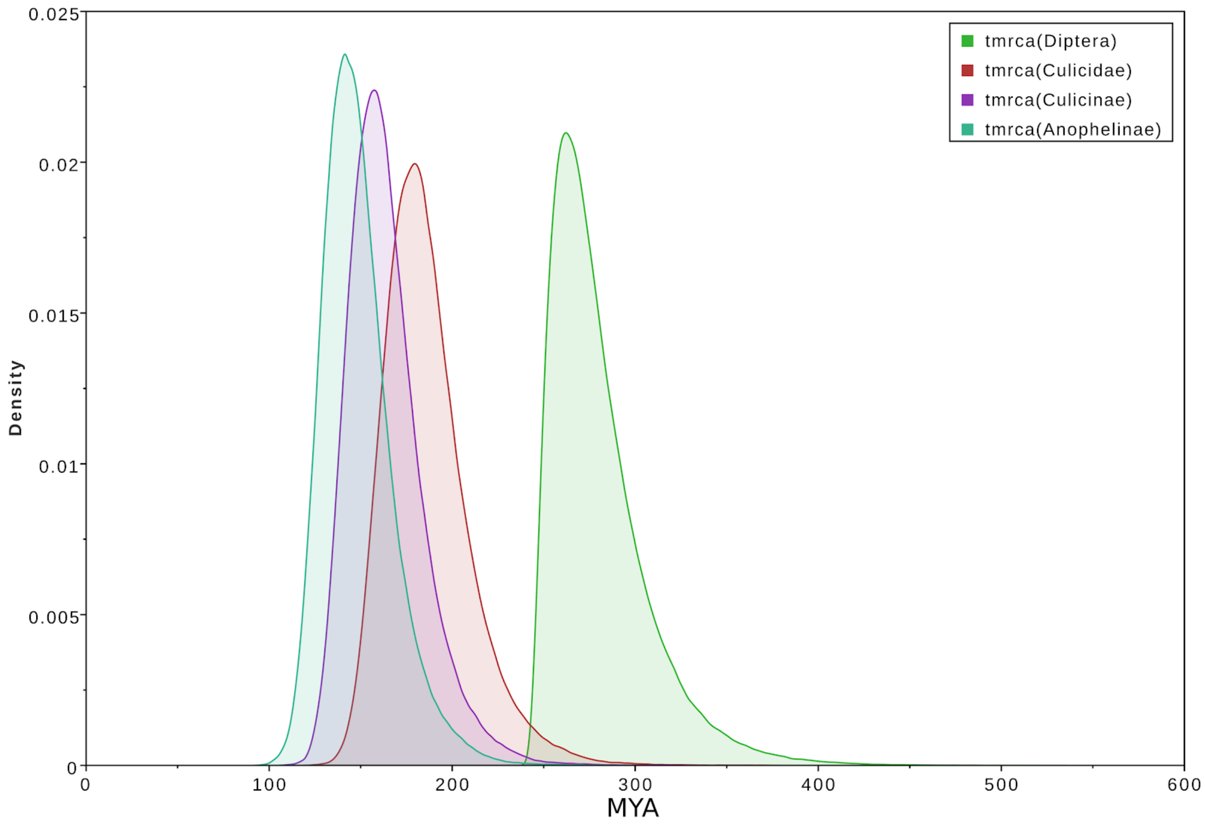

Supplementary figure 11. Density plot showing the 95% posterior distribution of ancestors time estimate based on the alignment of partitioned mitochondrial PCG with codon partition.

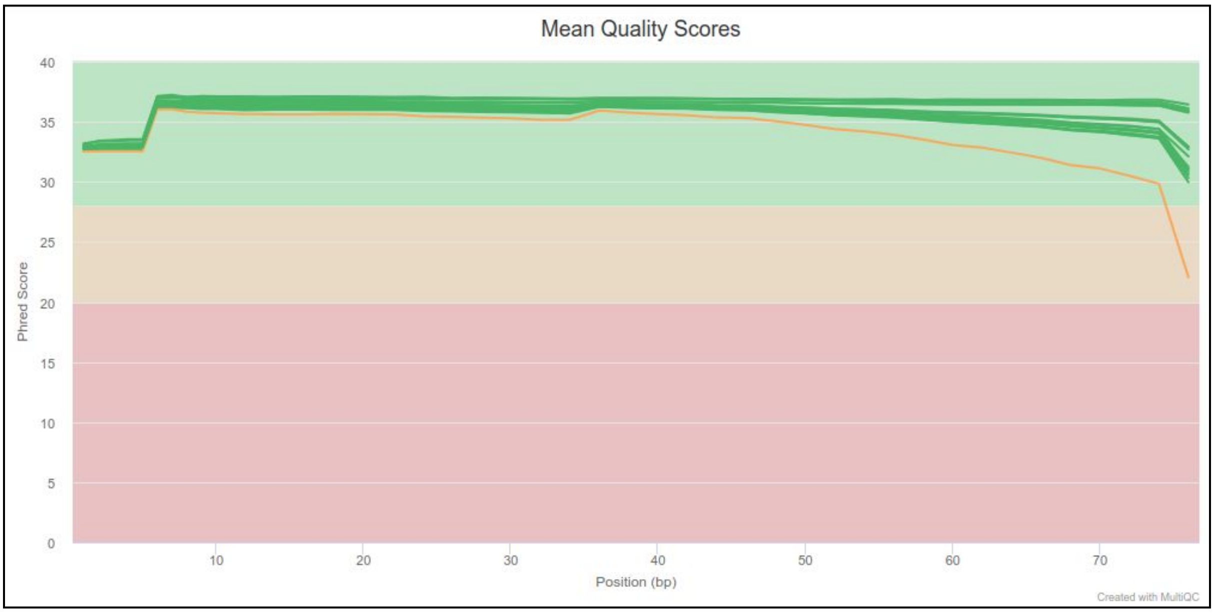

Supplementary figure 12. Quality of raw reads generated from mosquito samples. Figure generated from FasqQC results and summarized by MultiQC tool. The yellow line represents results from *Cq. albicosta* reads and green lines represent the remaining species.

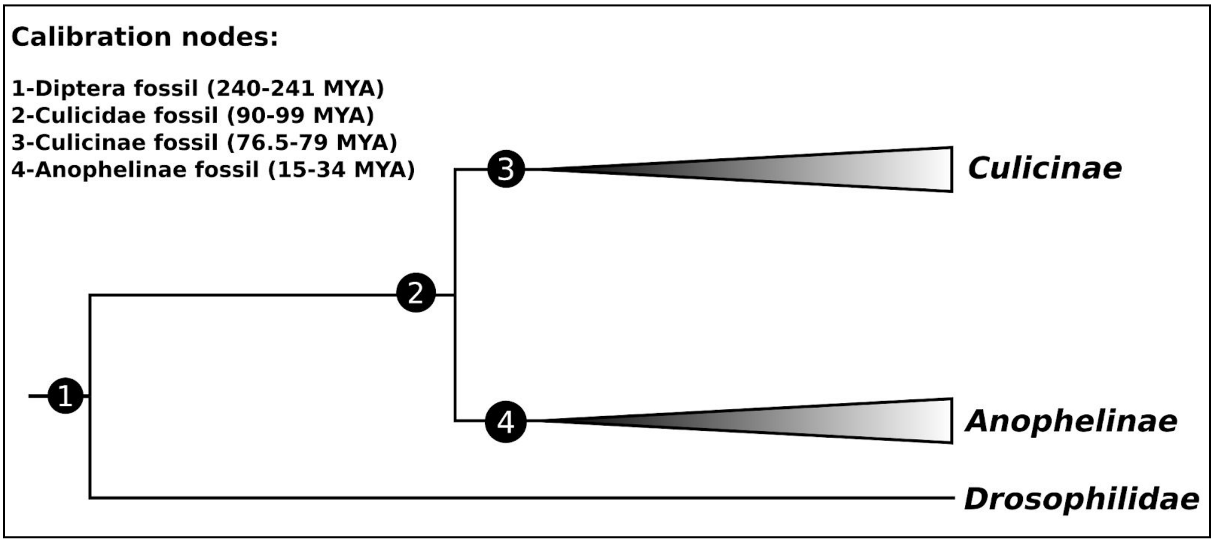

Supplementary figure 13. Schematic tree representing fossil dating used as calibration points priors in the molecular clock bayesian analyses. Circles with numbers inside represent each calibration point used based on the literature shown in Supplementary table 6.

References:

1. Behura, S. K. et al. Complete sequences of mitochondria genomes of *Aedes aegypti* and *Culex quinquefasciatus* and comparative analysis of mitochondrial DNA fragments inserted in the nuclear genomes. *Insect Biochem Mol Biol.* **41,** 770-777 (2011).
2. Hardy, C., Court, L., Morgan, M. & Webb, C. The complete mitochondrial DNA genomes for two lineages of *Aedes notoscriptus* (Diptera: Culicidae). *Mitochondrial DNA Part A.* **27**, 2024–2025 (2016).
3. Hardy, C., Court, L. & Morgan, M. The complete mitochondrial DNA genome of *Aedes vigilax* (Diptera: Culicidae). *Mitochondrial DNA Part A.* **27**, 2552–2553 (2016).
4. Krzywinski, J. et al. Analysis of the evolutionary forces shaping mitochondrial genomes of a Neotropical malaria vector complex. *Mol. Phylogenet. Evol.* **58**, 469–477 (2011).
5. Oliveira, T. et al. Mitochondrial Genomes of Anopheles (Kerteszia) (Diptera: Culicidae) From the Atlantic Forest, Brazil. *J. Med. Entomol.* **53**, 790–797 (2016).
6. Peng, X.-Y., Zhou, P., Duan, X.-Y. & Qian, Z.-Q. The mitochondrial genomes of twelve Anopheles mosquitoes (Diptera: Culicidae) and their phylogenetic implications. *Conserv. Genet. Resour.* **8,** 387–390 (2016).
7. Hua, Y.-Q. et al. Sequencing and analysis of the complete mitochondrial genome in *Anopheles culicifacies* species B (Diptera: Culicidae). *Mitochondrial DNA Part A.* **27**, 2909–2910 (2016).
8. Moreno, M. et al. Complete mtDNA genomes of *Anopheles darlingi* and an approach to anopheline divergence time. *Malaria Journal.* **9**, 127 (2010). doi.org/10.1186/1475-2875-9-127

9. Logue, K. et al. Mitochondrial genome sequences reveal deep divergences among *Anopheles punctulatus* sibling species in Papua New Guinea. *Malaria Journal*. **12**, 64 (2013). doi.org/10.1186/1475-2875-12-64

10. Krzywinski, J., Grushko, O. G. & Besansky, N. J. Analysis of the complete mitochondrial DNA from *Anopheles funestus*: An improved dipteran mitochondrial genome annotation and a temporal dimension of mosquito evolution. *Mol. Phylogenet. Evol.* **39**, 417–423 (2006).

11. Beard, C. B., Hamm, D. M. & Collins, F. H. The mitochondrial genome of the mosquito *Anopheles gambiae*: DNA sequence, genome organization, and comparisons with mitochondrial sequences of other insects. *Insect Mol. Biol.* **2**, 103–124 (1993).

12. Mitchell, S. E., Cockburn, A. F. & Seawright, J. A. The mitochondrial genome of *Anopheles quadrimaculatus* species A: complete nucleotide sequence and gene organization. *Genome.* **36**, 1058–1073 (1993).

13. Foster, P. G. et al. Phylogeny of anophelinae using mitochondrial protein coding genes. *R. Soc. Open Sci.* **4**, 170758 (2017).

14. Sun, L. et al. Sequencing and analysis of the complete mitochondrial genome of *Culex gelidus* (Diptera: Culicidae). *Mitochondrial DNA Part B.* **2**, 477–479 (2017).

15. Demari-Silva, B. et al. Mitochondrial genomes and comparative analyses of *Culex camposi, Culex coronator, Culex usquatus* and *Culex usquatissimus* (Diptera: Culicidae), members of the coronator group. *BMC Genomics.* **16**, 831 (2015).

16. Luo, Q.-C. et al. The mitochondrial genomes of *Culex tritaeniorhynchus* and *Culex pipiens pallens* (Diptera: Culicidae) and comparison analysis with two other Culex species. *Parasites & Vectors*. **9**, 406 (2016). doi.org/10.1186/s13071-016-1694-z

17. Lewis, O., Farr, C. & Kaguni, L. *Drosophila melanogaster* mitochondrial DNA: completion of the nucleotide sequence and evolutionary comparisons. *Insect Mol. Biol.* **4**, 263–278 (1995).
18. Lemos, P. et al. Characterization of mitochondrial genome of *Haemagogus janthinomys* (Diptera: Culicidae). *Mitochondrial DNA Part A.* **28**, 50–51 (2017).
19. Aragão, A. de O. et al. Description and phylogeny of the mitochondrial genome of *Sabethes chloropterus, Sabethes glaucodaemon* and *Sabethes belisarioi* (Diptera: Culicidae). *Genomics*. **111**, 607-611 (2019). doi:10.1016/j.ygeno.2018.03.016
20. Krzemiński, W., Krzemińska, E. & Papier, F. *Grauvogelia arzvilleriana* sp. n. - the oldest Diptera species (Lower/Middle Triassic of France). *Acta Zool. Cracov.* **37,** 95–99 (1994).
21. Borkent, A. & Grimaldi, D. A. The Earliest Fossil Mosquito (Diptera: Culicidae), in Mid-Cretaceous Burmese Amber. *Ann. Entomol. Soc. Am.* **97,** 882–888 (2004).
22. Poinar, G. O., Zavortink, T. J., Pike, T. & Johnston, P. a. *Paleoculicis minutus* (Diptera: Culicidae) n . gen ., n . sp ., from Cretaceous Canadian amber , with a summary of described fossil mosquitoes. *Acta Geol. Hisp.* **35,** 119–128 (2000).
23. Zavortink, T. J. & Poinar, G. O. *Anopheles (Nyssorhynchus) dominicanus sp*. n. (Diptera: Culicidae) from Dominican Amber. *Ann. Entomol. Soc. Am.* **93,** 1230–1235 (2000).
